# Pharmacokinetics and Pharmacodynamics of Perfluorooctane Sulfonate (PFOS) and Its Role in the Development and Progression of Prostate, Ovarian and Breast Cancers

**DOI:** 10.3390/cancers17213507

**Published:** 2025-10-31

**Authors:** Uche Okuu Arunsi, Daniel Chukwuebuka Ezirim, Chinonye Courage Arunsi, Ahmad Altayyar, Eke Godswill Uche, Favour Chidera Jonathan, Aluba Kalu Opieh, Ifeoma Vivian Anadi, Clinton Ositadinma Ofoegbu, Victor Chukwubuike Nwankwo, Eziuche Amadike Ugbogu, Paschal Emeka Etusim, Solomon Owumi

**Affiliations:** 1 School of Chemistry and Biochemistry, Georgia Institute of Technology, Atlanta, GA 30332-0400, USA; 2 Department of Biochemistry, Faculty of Biological Sciences, Abia State University, Uturu PMB 2000, Abia State, Nigeria; ezirimdaniel080@gmail.com (D.C.E.); ekegodswill05@gmail.com (E.G.U.); favourcjonathan@gmail.com (F.C.J.); aluziopieh96@gmail.com (A.K.O.); anadiifeoma6@gmail.com (I.V.A.); ofoegbuclinton1@gmail.com (C.O.O.); amadike.ugbogu@abiastateuniversity.edu.ng (E.A.U.); 3 Department of Nursing Science, Faculty of Health Sciences, Abia State University, Uturu PMB 2000, Abia State, Nigeria; chinonyecourage522@gmail.com; 4 Federal Medical Centre, Umuahia 440236, Abia State, Nigeria; 5 Naz Coker Ovarian Cancer Research Centre (NOVARC), Nottingham Biodiscovery Institute, School of Medicine, University of Nottingham, Nottingham NG7 2RD, UK; alyaa26@nottingham.ac.uk; 6 Department of Pharmaceutical Sciences, Irma Lerma Ramgel School of Pharmacy, Texas A&M University, College Station, TX 77843, USA; vnwankwo@tamu.edu; 7 Unit of Medical Parasitology and Environmental Entomology, Department of Animal and Environmental Biology, Faculty of Biological Sciences, Abia State University, Uturu PMB 2000, Abia State, Nigeria; paschalemeka.etusim@abiastateuniversity.edu.ng; 8 Cancer Research and Molecular Biology Laboratory, Department of Biochemistry, University of Ibadan, Ibadan 200005, Oyo State, Nigeria; zicri@hotmail.com

**Keywords:** perfluorooctane sulfonate, persistent organic pollutants, toxicokinetic, toxicodynamics, carcinogenesis, prostate cancer, breast cancer, ovarian cancer

## Abstract

Environmental pollution, driven by industrialization, urbanization, and agricultural practices, has intensified global ecological degradation. Among the most concerning pollutants is PFOS, a synthetic compound known for its chemical stability, environmental persistence, and bioaccumulative potential. Widely utilised in industrial and consumer products, PFOS infiltrates ecosystems and food chains, posing substantial risks to human and animal health. Upon exposure, PFOS disrupts lipid metabolism, damages cellular membranes, and alters signaling pathways through partial metabolism by cytochrome P450 enzymes. Accumulating evidence links PFOS to oxidative stress, mitochondrial dysfunction, endocrine disruption, neurotoxicity, and immunotoxicity. Critically, PFOS contributes to the development and progression of prostate, breast, and ovarian cancers via mechanisms such as hormonal interference, chronic inflammation, and epigenetic modifications. Epidemiological studies further associate elevated PFOS serum levels with increased cancer risk, particularly in occupationally and environmentally exposed populations. This review brings together the latest knowledge on PFOS emissions, mechanistic toxicity, and cancer-causing potential, highlighting the urgent need for focused research and improved regulatory measures to safeguard public health.

## Simple Summary

Perfluorooctane sulfonate (PFOS) is a synthetic chemical found in products like firefighting foams, stain-resistant fabrics, and food packaging. Due to its extreme stability, PFOS persists in the environment and accumulates in living organisms, including humans. PFOS can enter the ecosystems and human bodies, where it has been linked to hormone disruption, DNA damage, and immune and metabolic disturbances. Most concerning is PFOS’s potential to cause cancers of the prostate, breast, and ovary. This review highlights the need for urgent global measures to limit PFOS exposure and safeguard public health.

## 1. Introduction

**Figure 1:**
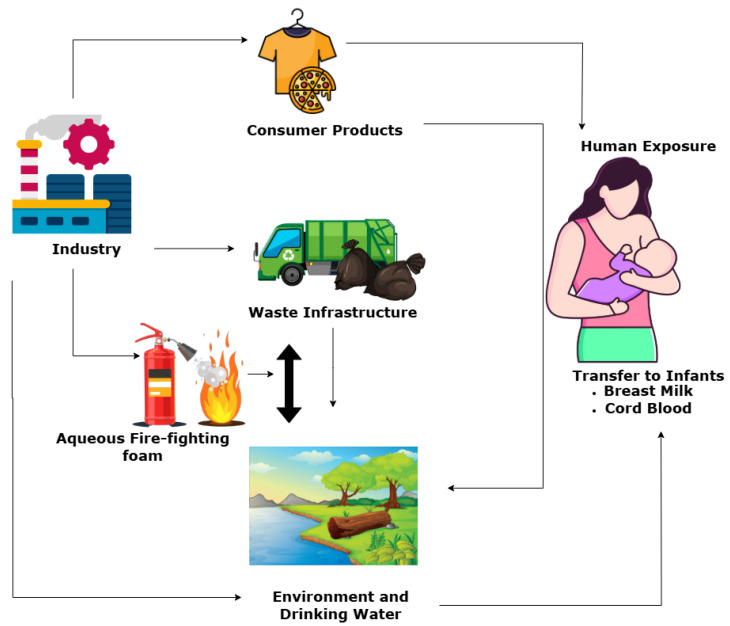
Primary exposure routes of PFOS. PFOS enters the environment through industrial activities, consumer products, aqueous fire-fighting foams, and waste infrastructure. Contaminated water and ecosystems serve as major pathways for human exposure, including maternal transfer via breast milk and cord blood. Arrows indicate the directional flow of PFOS from sources to exposure endpoints. Source: Generated with https://app.diagrams.net/, accessed on 23 April 2025.

Environmental pollution, caused by human activities such as industrial processes, farming, urbanization, and changes in land use, as well as transport, fossil fuel use, poor waste disposal, mining, and the production of household and consumer goods, has led to widespread environmental damage worldwide. This damage is also influenced by natural factors such as wildfires, volcanic eruptions, soil erosion, desertification, tsunamis, storm surges, natural gas emissions, and ocean and atmospheric circulation patterns. PFOSs represent a class of synthetic perfluorinated compounds widely acknowledged due to their remarkable stability, hydrophobicity, and surfactant properties. PFOS has found extensive application in industrial and consumer sectors, including stain-resistant fabrics, firefighting foams, metal plating, and semiconductor manufacturing [1,2,3]. Owing to its distinctive chemical structure, which is characterized by a fully fluorinated carbon chain in conjunction with a sulfonate functional group, PFOS displays exceptional resistance to degradation [4,5], thereby rendering it a significant concern for environmental and public health. PFOS, classified as a persistent organic pollutant (POP), exhibits substantial ecological persistence, bioaccumulation potential, and capability for long-range transport [6]. It shows resistance to conventional degradation processes such as advanced oxidation methods [7,8,9], reductive degradation methods [10], biodegradation and microbial degradation [11,12], and thermal decomposition and incineration [13,14], leading to extensive contamination of atmospheric, aquatic, terrestrial, and biological systems [4,15]. PFOSs are primarily detected in water bodies such as rivers, estuaries, and oceans, and they are closely associated with anthropogenic activities and natural influences. Their integration into the food chain and aquatic systems has culminated in their presence within food and water supplies, consequently leading to chemical risks associated with dietary exposure (**Figure 1**). Research findings have established connections between PFOS exposure and conditions such as oxidative stress, mitochondrial dysfunction, endocrine disruption, neurological dysfunction, and immunotoxic effects [16,17,18,19], which raises significant concerns regarding its protracted implications for both human and ecological health. Given its propensity to accumulate in hepatic and plasma tissues [20,21,22], PFOS has been detected globally in various wildlife species, marine organisms, and human populations.

**Figure 2:**
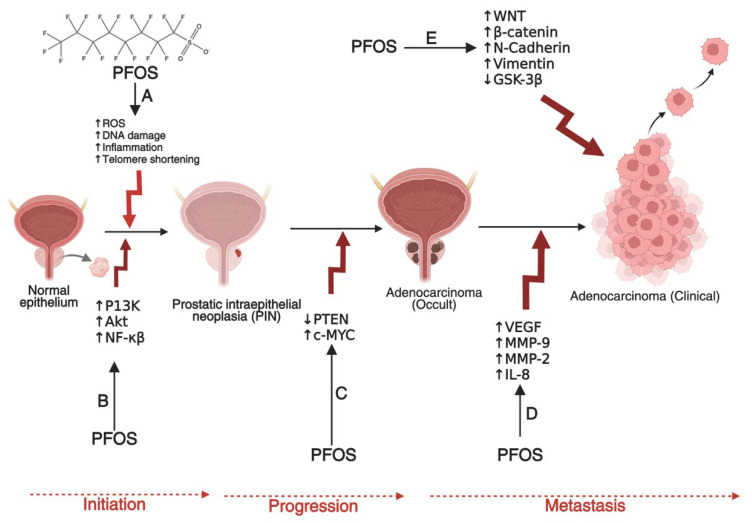
PFOS-mediated induction and progression of PCa. PFOS mediates the transition from normal prostate epithelium to clinical adenocarcinoma. **A**: The mechanisms of tumor initiation by PFOS involve oxidative stress, DNA damage, inflammation, and telomere shortening, leading to prostatic intraepithelial neoplasia (PIN). **B**: The formation of PIN can be driven by the activation of other signaling nodes, such as PI3K/Akt/NF-κB. **C**: Progression is driven by activation of oncogenes such as c-MYC and the repression of PTEN by PFOS. **D**: PFOS-mediated increase in the expression of IL-8, MMP-2, MMP-9, and VEGF then mediates the transformation of occult adenocarcinoma to clinical adenocarcinoma. **E**: Other signaling pathways, such as WNT/β-catenin, N-Cadherin and vimentin further facilitate the metastasis of PCa to distant organs Generated with BioRender (https://app.biorender.com, accessed on 28 October 2025).

**Figure 3:**
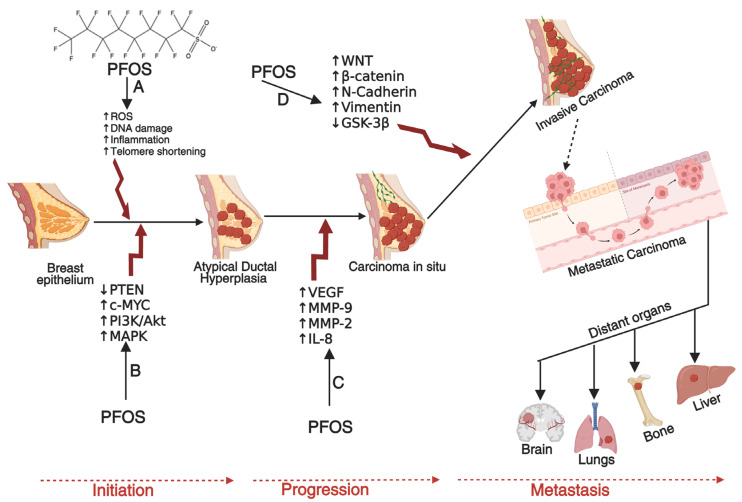
PFOS-mediated induction and progression of BCa. PFOS drives the sequential transformation of normal breast epithelial cells into metastatic carcinoma. **A**: The initiation step involves DNA damage, inflammation, and telomere shortening. **B**: These processes can deregulate the expression of certain genes, such as PTEN, c-MYC, PI3K/Akt, and MAPK, leading to the formation of atypical ductal hyperplasia. **C**: The mechanisms of progression are marked by elevated expression of VEGF, MMP-2/9, and IL-8 levels, which promote tumor growth and angiogenesis, leading to the formation of carcinoma in situ. **D**: PFOS can further trigger the transformation of carcinoma in situ into invasive carcinoma. This process is facilitated by WNT/β-catenin signaling and epithelial–mesenchymal transition markers, enabling dissemination to distant organs such as the lungs, liver, bones, and brain. Generated with BioRender (https://app.biorender.com, accessed on 28 October 2025).

**Figure 4:**
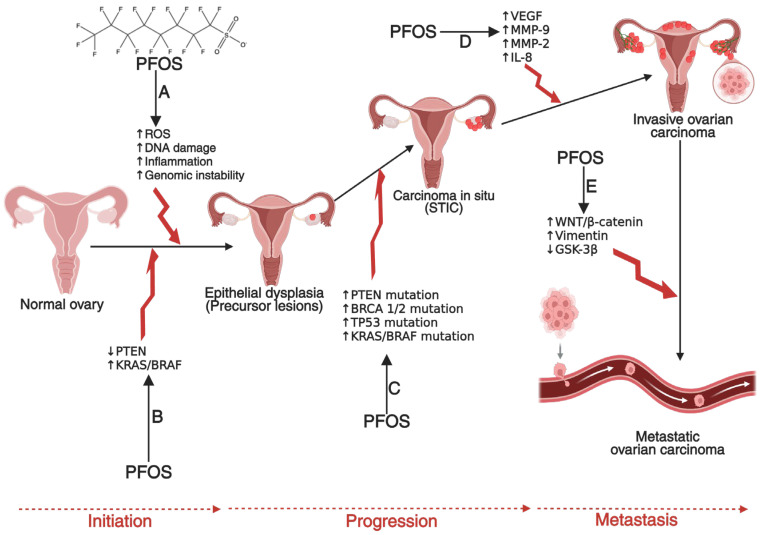
PFOS-mediated induction and progression of OC. Exposure to PFOS mediates the transformation of normal ovarian tissues into metastatic ovarian carcinoma. **A**: The Initiation step involves oxidative stress, DNA damage, inflammation, and genomic instability. **B**: These biological processes can induce dysregulation in the expression of some genes, including PTEN, KRAS and BRAS, thus leading to epithelial dysplasia (precursor lesions). **C**: The progression step is driven by the accumulations of several mutations, including PTEN, BRCA 1/2, TP53, KRAS, and BRAS, ultimately resulting in the formation of carcinoma in situ (otherwise known as serous tubal intraepithelial carcinoma (STIC)). **D**: PFOS further drives the activation of several genes, including VEGF, MMP-9/MMP-2, and IL-8. This process is necessary for tumor angiogenesis and invasion, leading to the formation of invasive ovarian carcinoma. **E**: The metastatic step is facilitated by WNT/β-catenin signaling, vimentin upregulation, and GSK3β inhibition, enabling epithelial–mesenchymal transition and cellular migration. Generated with BioRender (https://app.biorender.com, accessed on 28 October 2025).

Carcinogenesis is a multi-step process involving genetic mutations, epigenetic modifications, and typical cellular regulatory pathway disruptions [23,24,25]. PFOS has been found to drive these processes through multiple mechanisms. One key mechanism is endocrine disruption, where PFOS mimics or alters the activity of androgen hormones. In breast and ovarian cancers, PFOS has been shown to enhance estrogen receptor (ER) signaling, leading to increased proliferation of mammary gland cells and ovarian epithelial cells, which may contribute to the development of breast and ovarian cancers if left unchecked [26,27,28,29]. Similarly, in prostate cancer (PCa), PFOS has been shown to disrupt androgen receptor (AR) pathways, promoting abnormal cell growth and progression of PCa [30,31,32]. In addition to hormonal interference, PFOS contributes to carcinogenesis by inducing oxidative stress through the persistent generation of reactive oxygen species (ROS) [33,34]. Increased ROS levels can cause DNA damage, impair DNA repair mechanisms, and trigger inflammatory responses that create a tumor-promoting microenvironment [35,36,37,38]. Chronic inflammation plays a critical role in the development and progression of cancers by sustaining cellular proliferation, preventing apoptosis, and facilitating metastasis [39,40,41]. Other mechanisms that promote the development and progression of prostate, breast and ovarian cancers by PFOS include down-regulation of PTEN and GSK-3-β, as well as activation of PI3K/Akt/NF-κB signalling node, c-MYC, WNT1, β-catenin, N-Cadherin, Vimentin, VEGF, MMP-2/9, and pro-inflammatory cytokines and chemokines [42,43]. In PCa, these molecular alterations can disrupt the normal cellular and genetic architecture of prostate epithelial cells, leading to the development of prostatic intraepithelial neoplasia (PIN) in prostate tissues (**Figure 2**), promoting the transformation of normal breast epithelial cells into malignant phenotypes with enhanced migratory potential to distant organs such as the brain, lungs, bones, and liver (**Figure 3**), or enhancing the transformation of ovarian epithelial cells to metastatic ovarian cancer (OC) (**Figure 4**). Furthermore, an upregulation of several metastatic markers, including VEGF, MMPs, and the WNT/β-catenin pathways, will further coordinate the progression of clinical prostate, breast, and ovarian cancers [42,43,44]. Epigenetic modifications are another pathway through which PFOS may drive cancer development. Studies have shown that PFOS exposure alters DNA methylation patterns and histone modifications, leading to the suppression of tumor suppressor genes and the activation of oncogenes [45,46,47]. These genetic and epigenetic changes contribute to uncontrolled cell division and progression of PCa and breast cancer (BCa) [48,49].

Epidemiological studies have provided further support for the link between PFOS exposure and cancers. Research has found that individuals with higher serum PFOS levels have an increased risk of developing prostate, ovarian and breast cancers, particularly in populations with occupational or environmental exposure [32,50,51]. While more research is needed to establish direct causation, the accumulating evidence underscores the need for stricter regulations on PFOS production and use to mitigate potential health risks. Given its widespread environmental presence and long-term persistence in biological systems, PFOS poses a significant public health concern. Understanding the molecular and cellular mechanisms through which PFOS contributes to PCa development and progression is crucial for guiding future research, informing regulatory policies, and implementing strategies to reduce exposure. We are currently examining the toxicological effects of PFOS, concentrating on its emission and environmental distribution, the mechanistic toxicities impacting both human and ecological health, and its potential involvement in the development of prostate, ovarian and breast cancers.

## 2. Emission and Environmental Distribution of PFOS

**Figure 5:**
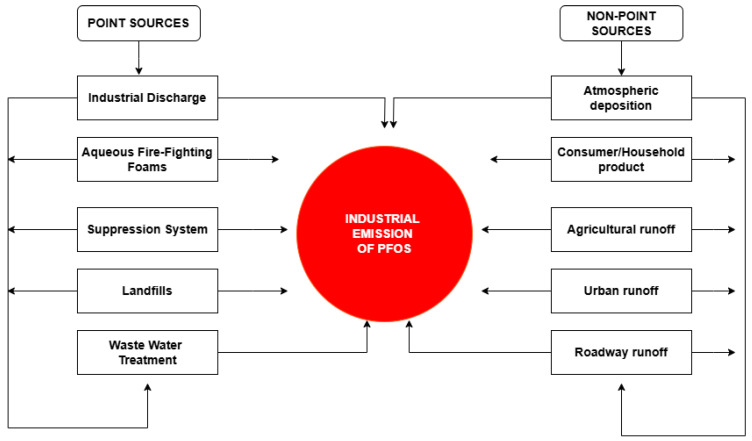
Emission and distribution pathways of PFOS. Point sources include industrial discharge, aqueous fire-fighting foams, suppression systems, landfills, and wastewater treatment facilities. Non-point sources encompass atmospheric deposition, consumer and household products, agricultural runoff, urban runoff, and roadway runoff. Arrows indicate the flow of PFOS from emission sources into the environment. Generated with https://app.diagrams.net/, accessed on 28 April 2025.

Due to its chemical stability, resistance to degradation, and extensive industrial applications, PFOS can enter the environment through two principal sources (**Figure 5**): point sources and nonpoint sources [52,53,54,55]. Point sources are defined as identifiable and confined sources of pollution from which PFOS is discharged directly into the air, water, or soil. Because they originate from specific locations, this source type can be easily monitored and regulated. Illustrative examples of point sources of PFOS include industrial discharges, firefighting and fire suppression systems, landfills, and wastewater treatment plants [56,57]. Chemicals containing PFOS have been employed in the treatment of high-quality textiles as finishing agents, often affecting their water, oil, and soil resistance characteristics. The PFOS chemicals utilized are PFOS polymers with a residual PFOS content of 1%. Textile formulations contain, on average, around 27% PFOS polymers. Additionally, back-coating textiles can result in losses, with an emission factor as outlined in the Emission Scenario Document [58]. Recent studies have shown that fluorochemical production plants (FPPs) emit dozens of PFOS [59] based on wastewater analysis. The production of fluoropolymers and their livestock substances is intrinsically related to the use, formation, and emission of PFOS. This has led to high PFOS exposure in people near these FPPs. Companies such as 3M and DuPont, which released significant quantities of PFOS, were cognizant of the toxicity associated with PFOS and the detection of fluorochemicals in workers’ bloodstreams by the 1970s [60]. In the production of aqueous firefighting foams (AFFF), PFOS chemicals have been predominantly employed for fire protection in petrochemical industries, fire departments, and military establishments. The United States Air Force has identified approximately 200 installations where PFOS was used, demonstrating that the United States military ranks among the top consumers of AFFF [61]. In China, the consumption of PFOS-related chemicals is also substantial, amounting to approximately 100 tons annually, with over 50 enterprises engaged in the manufacturing of AFFF [58]. The release of AFFF results in the contamination of soil and water sources with PFOS, particularly in the vicinity of airport firefighting training facilities and fire stations. The significant risk of agricultural contamination caused by PFOS has been monitored and studied [62]. Wastewater treatment plants (WWTPs) are the primary studied point sources of PFOS in surface waters, and they can serve as perennial sources of PFOS to the environment, including biosolids [61]. Biosolids in wastewater treatment plants can be used in agriculture. Biosolids contain large amounts of nitrogen, phosphorus, and organic carbon. Therefore, they are applied as soil amendments [62]. Studies have shown that the extensive application of wastewater biosolids in soil is a pollution pathway of PFOS in agricultural plants [62].

Moreover, nonpoint sources pertain to dispersed and indirect origins of pollution, particularly in contexts where PFOS contamination is observed across vast areas. Nonpoint sources include atmospheric deposition, household and consumer product use, agricultural runoff and urban and roadway runoff [54,63,64]. Such sources present more significant challenges in terms of monitoring and regulation. One notable nonpoint source of PFOS is atmospheric deposition. PFOS and associated perfluorinated compounds are emitted into the atmosphere via industrial emissions, combustion processes, and volatilization from treated surfaces. These airborne contaminants can traverse extensive distances before settling into aquatic systems, soil, and vegetation through precipitation or dry deposition mechanisms [65,66]. D’Ambro [55], in comparing predicted per- and polyfluoroalkyl substances (PFAS) and GenX air concentrations from a fluorochemical manufacturing facility in Eastern North Carolina, United States, found that the area surrounding the facility experiences around 15 days per year of GenX concentration above the inhalation screening level they derived in their study. Household and consumer products represent another primary nonpoint source of PFOS. Many everyday items, including stain-resistant textiles, carpets, nonstick cookware, and food packaging, contain PFOS-based treatments. Over time, the wear and degradation of these products release PFOS into indoor dust, wastewater, and the broader environment. Washing treated fabrics and carpets leads to PFOS contaminating sewage, eventually reaching surface waters through wastewater treatment plants [67,68].

There is substantial evidence indicating that PFOS may accumulate in edible fish species to levels of up to 7 and 170 ng/g wet weight (wwt). Notably, the Minnesota Department of Health has issued advisories concerning fish consumption in areas of the Mississippi River affected by contamination [68]. The land application of biosolids and landfill leachate significantly contributes to nonpoint source contamination by PFOS. Wastewater treatment facilities accept and process effluent from residential, commercial, and industrial origins; however, they do not eliminate PFOS. As a result, the treated wastewater released into fluvial and lacustrine environments acts as a persistent nonpoint source of PFOS within aquatic ecosystems [69]. Likewise, biosolids produced from wastewater treatment are frequently used as agricultural fertilizers. Since biosolids contain residual PFOS, their application can result in PFOS accumulation in the soil, posing a risk of crop contamination and subsequent entry into the food chain [70]. Runoff from contaminated sites is another widespread nonpoint source. Areas with historical PFOS use, such as firefighting training grounds, airports, and military bases, often have residual contamination in soil and groundwater. PFOS can leach from these sites into surrounding water bodies during rainfall, leading to diffuse pollution [71,72,73]. Contamination of marine and freshwater through bioaccumulation also contributes to nonpoint PFOS pollution. PFOS is extremely persistent and accumulates in aquatic organisms, resulting in biomagnification within the food web. Contaminated fish and seafood are secondary nonpoint sources when consumed by humans and other animals, redistributing PFOS throughout ecosystems [74]. Overall, the complexity of nonpoint PFOS sources highlights the challenge of managing environmental contamination. Unlike point sources, which can be specifically regulated, nonpoint sources require a combination of monitoring, pollution mitigation, and policy measures to reduce their impact on human health and ecosystems.

## 3. Mechanisms of PFOS Toxicokinetics and Dynamics

### 3.1. Absorption and Distribution of PFOS in Human Tissues and Organs

Humans are exposed to PFOS through various routes such as contaminated drinking water, food intake, consumer goods, and occupational contact [75]. Currently, no known families of enzymes are recognized to metabolize PFOS; therefore, it is classified as a “forever chemical”. Nevertheless, recent research indicates that ingestion of PFOS may inhibit the activity of the CYP540 enzyme family, including enzymes such as CYP1A2, CYP2B6, CYP2C19, CYP2E1, and CYP3A4 [76]. Once in the body, PFOS exhibits unique pharmacokinetic characteristics that set it apart from conventional persistent organic pollutants (POPs) [77]. PFOS has a strong affinity for proteins, especially serum albumin and liver fatty acid-binding proteins. This protein-binding behavior promotes their persistence and accumulation in protein-rich organs [78]. The first study to measure PFOS levels in human internal tissues (excluding blood) was by Olsen et al. [79], who detected PFOS in serum and liver samples from non-exposed individuals. The mean PFOS levels were 18.8 ng/g in liver and 17.7 ng/mL in serum, with a liver-to-serum ratio of 1.3:1. Maestri et al. [80] later analyzed pooled organ samples from seven unexposed individuals in Italy, detecting PFOS across multiple organs, with the highest concentrations in the liver, kidney, and lungs. A German pilot study by Völkel et al. [81] reported mean liver concentrations of 17.9 ng/g of PFOS. These early studies collectively highlighted PFOS accumulation in protein-rich organs, particularly the liver, even among non-occupationally exposed individuals. In a later study, Liu et al. [82] analyzed PFOS in tumor and non-tumor liver tissues from liver cancer patients in China. The mean PFOS levels were comparable between non-tumour liver tissues (22.2 ng/g) and tumor liver tissue (22.4 ng/g) samples, indicating widespread PFOS accumulation in both healthy and malignant liver tissues. In Pavia, Italy, Pirali et al. [83] measured PFOS levels in thyroid tissues from 28 patients who underwent surgery for benign or malignant thyroid disorders. PFOS was detected in all thyroid samples, with median concentrations of 5.3 ng/g (range: 2.1–44.7 ng/g). In addition, Mamsen et al. [84] analyzed PFOS in placenta and fetal tissues from legally terminated pregnancies in Denmark. They found the highest concentrations of 1.3 ng/g in the placenta. They also examined PFOS in embryos, placentas, and multiple fetal organs (liver, lung, heart, CNS, and adipose tissue). PFOS showed the highest median concentrations in both placenta (1.24 ng/g) and fetal tissues (0.83 ng/g). These findings confirmed that PFOS can cross the placenta and accumulate in fetal tissues, raising concerns about prenatal exposure risks.

### 3.2. PFOS–DNA Interaction Through Non-Covalent Binding

The primary pathways of PFOS toxicity involve non-covalent interactions with DNA and proteins, as demonstrated by both experimental and computational evidence [85,86]. These interactions are subtle yet biologically significant, providing a plausible mechanistic link between PFOS exposure and genomic instability observed in mammalian systems. The interaction of PFOS with DNA is principally influenced by electrostatic forces, hydrogen bonds, and hydrophobic interactions. The sulfonate head group of PFOS possesses a substantial negative charge, enabling it to establish electrostatic associations with cationic regions of DNA-binding proteins or via divalent metal-mediated bridges such as Mg^2+^ or Ca^2+^ in proximity to the DNA phosphate backbone. Concurrently, the perfluorinated carbon chain engages in hydrophobic and van der Waals interactions with nucleobases, notably in adenine-thymine (A–T) rich regions, thereby facilitating its insertion into the minor groove of DNA and contributing to the stabilisation of PFOS–DNA complexes [87].

Spectroscopic analyses, including UV–Vis absorption, fluorescence quenching, and circular dichroism, have demonstrated changes in DNA secondary structure upon PFOS binding. These conformational distortions are characterized by a reduction in base stacking and an alteration of the helical twist, indicative of PFOS-induced perturbations in DNA stability and Molecular docking simulations further corroborate these observations, showing that PFOS can localize within DNA grooves, where its fluorinated tail aligns parallel to the helical axis, while the sulfonate group interacts with polar residues, creating a thermodynamically stable but conformationally disruptive complex [87]. Functionally, such interactions can have profound biological consequences. PFOS–DNA binding may hinder the access of DNA polymerases and repair enzymes to their substrates, resulting in replication stress, transcriptional interference, and impaired DNA repair fidelity. These disturbances are consistent with empirical findings that PFOS exposure induces DNA strand breaks, micronuclei formation, and oxidative stress-mediated genotoxicity in mammalian cells. Moreover, epigenetic alterations such as aberrant DNA methylation and histone modification patterns have been reported, suggesting that PFOS not only affects the physical integrity of DNA but also modulates chromatin structure and gene expression [88].

### 3.3. Disruption of Lipid Metabolism and Mitochondrial Function

**Figure 6:**
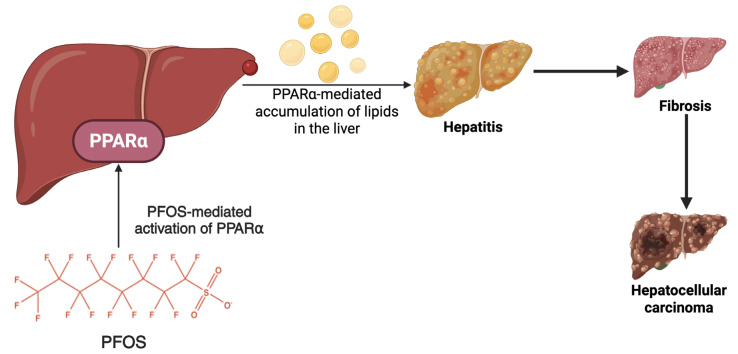
PFOS-mediated disruption of lipid metabolism in the liver. PFOS activates peroxisome proliferator-activated receptor alpha (PPARα), leading to lipid accumulation in hepatic cells. This dysregulation progresses from hepatic steatosis (fatty liver) to inflammation (hepatitis), fibrosis, and ultimately hepatocellular carcinoma. Generated with BioRender (https://app.biorender.com, accessed on 28 April 2025).

The liver is the primary organ for fatty acid metabolism and transport. It plays a central role in regulating lipid homeostasis through various metabolic pathways. Numerous metabolic alterations have been linked to PFOS exposure. It has been reported that PFOS (**Figure 6**) can activate peroxisome proliferator-activating receptor alpha (PPARα), crucial in regulating lipid metabolism and adipogenesis [89]. PFOS can disrupt the balance of lipid metabolism in the liver, resulting in a series of pathological changes [90] and consequently leading to fatty liver, liver fibrosis, and hepatocellular carcinoma [91]. At near cytotoxic concentrations, PFOS induces the accumulation of triglycerides in HepaRG cells [92] and consistently affects the expression of steatosis marker genes and target genes for PPAR-α [93]. Studies have also revealed that mitochondria function in the hepatocytes [94], oocytes [95,96], and pancreatic β cells [97] can suffer alterations due to PFOS exposure. Hoffman et al. [98] reported that PFOS caused a concentration-dependent reduction in basal, maximal, and ATP-linked respiration in the mitochondria, thereby disrupting placental function. By compromising the bioenergy capacity of trophoblasts, PFOS exposure causes a decline in mitochondrial content and impairs cellular respiration, leading to mitochondrial dysfunction. The mitochondria’s transmembrane potential (ΔΨm) depends on the cellular oxidative phosphorylation. Depolarization of ΔΨm, perhaps caused by mitochondrial swelling, will produce lower ATP [99]. Prenatal exposure to PFOS may have the potential to induce cardiac mitochondrial injury and apoptosis [100].

### 3.4. Oxidative Stress and Reactive Oxygen Species (ROS) Generation

**Figure 7:**
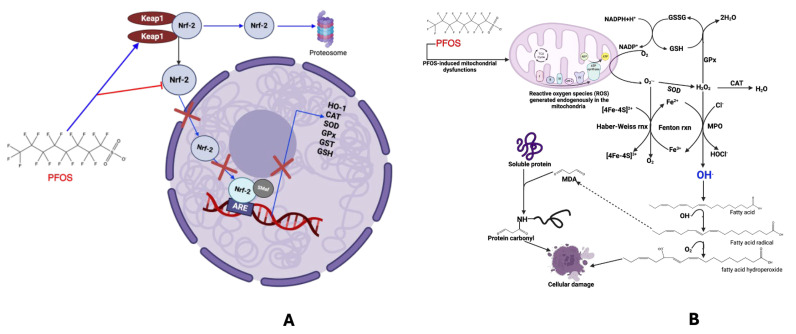
PFOS-mediated induction of oxidative stress. (**A**): PFOS disrupts the Keap1–Nrf2 signaling axis, promoting the nuclear translocation of Nrf2 and its binding to antioxidant response elements (AREs), which activates transcription of cytoprotective genes. (**B**): PFOS enhances mitochondrial production of reactive oxygen species (ROS), including superoxide anions (O_2_^−^), hydrogen peroxide (H_2_O_2_), and hydroxyl radicals (OH•), leading to oxidative damage of lipids, proteins, and DNA. These events contribute to cellular dysfunction and apoptosis. Generated with BioRender (https://app.biorender.com, accessed on 4 May 2025).

Several studies have revealed that oxidative responses are associated with PFOS-induced cell injury and tissue damage. The Keap-1/Nrf-2 signaling axis has been recognized as one of redox biology’s most intriguing and complex pathways [36,101,102]. Serving as a cellular sensor for oxidative stress, this pathway exemplifies nature’s strategic design in safeguarding cells against the incessant onslaught of ROS. By meticulously regulating the transcriptional activity of Nrf-2, Keap1 functions as a molecular gatekeeper, permitting the stabilization of Nrf-2 solely when the redox equilibrium is disrupted, thereby ensuring a swift yet controlled antioxidant response. Under physiological conditions, the Keap-1/Nrf-2 system is pivotal, activating many cytoprotective genes that coordinate antioxidant defence, detoxification, and metabolic adaptation. Specifically, Nrf-2 moves into the nucleus, where it works together with other transcription factors to turn on genes like heme oxygenase-1 (HO1), catalase (CAT), glutathione peroxidase (GPX), glutathione*-s-*transferase (GST), and a variety of other helpful protective genes. This response is especially critical in tissues subjected to high oxidative stress, including the lungs, liver, and skin. However, in the presence of toxicants such as PFOS, Keap-1 activation is more favored, leading to the repression of Nrf-2 (**Figure 7A**). In this circumstance, Nrf-2 is targeted for degradation by the proteasomal system, thereby lowering the GSH, GST, and HO-1 [103]. PFOS induction of oxidative stress is achieved via inhibition of antioxidants (e.g., superoxide dismutase, catalase, GPx4, and GSH) or enhancing the generation of ROS in hepatocytes or endothelial cells will lead to cell death [33,104,105,106,107]. Daenen et al. [108] also reported that oxidative stress produces ROS, causing oxidative damage to nucleic acids, proteins, and lipids, ultimately leading to epithelial cell death and a decline in kidney function (**Figure 7B**). PFOS induces apoptosis in pulmonary cells via the reactive oxygen species-mediated mitochondria-dependent pathway [109]. Lee et al. [110] and Wen et al. [111] suggested that oxidative stress and mitochondrial function are pivotal in renal tubular cell apoptosis induced by PFOS. In the liver, PFOS exposure can increase ROS production and disrupt the intracellular antioxidant defense system [112]. Production of ROS in excess is cytotoxic, resulting in oxidative damage to hepatocyte biomolecules [113]. By disrupting the electron transport chain and releasing cytotoxic reactive oxygen species (ROS), PFOS can directly inflict damage on the mitochondria of hepatic cells, leading to the production of hydrogen peroxide (H_2_O_2_). This compound subsequently diffuses into the lysosome, resulting in the formation of highly reactive hydroxyl radicals [114]. The accumulation of H_2_O_2_ and the elevated MDA content induced by PFOS exposure will result in a reduction in SOD and GSH levels [115]. Excess ROS may compromise the integrity of hepatocyte cell membranes, thereby affecting their stability and permeability [116]. ROS can disrupt calcium homeostasis by affecting calcium channels, pumps, and storage organelles, increasing intracellular calcium levels. This dysregulation contributes to cell damage and apoptosis, particularly in hepatocytes [117].

### 3.5. DNA Damage and Epigenetic Modification

**Figure 8:**
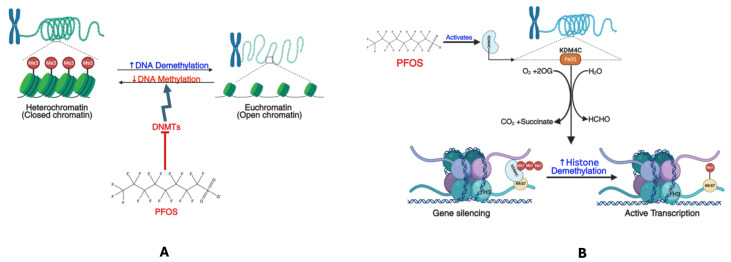
PFOS-mediated epigenetic modifications. (**A**): PFOS disrupts DNA methylation dynamics by inhibiting DNA methyltransferases (DNMTs) and promoting demethylation, leading to the transition from transcriptionally repressive heterochromatin to active euchromatin and aberrant gene expression. (**B**): PFOS alters histone methylation through the upregulation of histone demethylases, affecting chromatin structure and transcriptional activity. These changes contribute to the activation of oncogenes and suppression of tumor suppressor genes. Generated with BioRender (https://app.biorender.com, accessed on 4 May 2025).

Another PFOS toxicity mechanism is genetic and epigenetic perturbations (**Figure 8**). Genetic damage, including the breakage of DNA strands, DNA fragmentation, and chromosomal breaks, results from PFOS toxicity [118]. Exposure to PFOS was found to cause direct oxidative damage [119], increase ROS generation, and subsequently lead to DNA damage in liver cancer cell lines (HepG2) [120]. In peripheral blood, Eke and Celik [121] reported that doses of PFOS increase the micronucleus frequency and strongly induce DNA damage. Gonadal development is also impaired by PFOS, leading to a decline in the total count of germ cells, transient arrest in the mitotic cycle, and apoptosis via DNA damage induced by ROS [122]. PFOS exposure distorts proteome structure, aggravates oxidative stress, compromises mitochondrial oxidative phosphorylation, enhances mitochondrial permeability, and promotes DNA damage and apoptosis [18].

Findings from epigenetic studies demonstrate how PFOS influences gene expression without altering DNA sequence [123]. Different epidemiological studies assessed via Infinium arrays have identified that prenatal PFOS exposure is associated with variations in newborn or childhood blood methylation [99,124,125]. Kim et al. [126] found a significant link between PFOS and epigenetic modification in both adult and newborn cohorts. These modifications can activate oncogenes and/or inhibit tumor suppressor gene expression through DNA methylation, histone modification, and non-coding RNAs [127]. Research suggests that PFOS can contribute to cancer development by altering gene expression and triggering epigenetic changes. This, in turn, affects multiple signaling pathways in various cell types [128]. In the study of Wen et al. [129], exposure to PFOS was found to disrupt the epigenetic signatures, resulting in global DNA hypomethylation and upregulation of histone lysine demethylases, specifically KDM1A (LSD1) and KDM4C (JMJD2C). These alterations contribute to modified gene expression patterns, genomic instability, and increased disease vulnerability. DNA methylation, an essential epigenetic mechanism, entails adding methyl groups (-CH3) to cytosines within CpG dinucleotides, usually leading to gene silencing [130,131,132]. PFOS exposure has been correlated with a worldwide decline in DNA methylation (**Figure 8A**), which may stem from several mechanisms: the inhibition of DNA methyltransferases (DNMTs), disruption of one-carbon metabolism, and the activation of DNA demethylases [129,133,134,135]. In inhibiting DNMTs, PFOS might diminish the expression and activity of DNMT1, DNMT3A, and DNMT3B, enzymes accountable for maintaining and establishing DNA methylation patterns. This ultimately leads to a gradual loss of methylation across the genome, especially in tumour suppressor genes and repetitive DNA elements, thereby causing increased genomic instability. Regarding one-carbon metabolism disruption, PFOS may hinder S-adenosylmethionine (SAM) production, the primary methyl donor for DNA methylation, and lower SAM levels adversely affect de novo methylation, further facilitating hypomethylation. The activation of DNA demethylases by PFOS is linked to enhanced activity of TET enzymes (Ten-Eleven Translocation proteins), which catalyze the oxidation of 5-methylcytosine (5mC) to 5-hydroxymethylcytosine (5hmC), thereby initiating DNA demethylation. As an outcome of these transformations, oncogenes become abnormally activated, while tumour suppressor genes lose their repressive function, fostering uncontrolled cell proliferation and cancer progression [129,136,137].

KDMs are critical in remodeling chromatin and regulating gene expression [138,139,140]. PFOS has been demonstrated to elevate the expression levels of KDM1A and KDM4C [129], two principal histone demethylases that erase repressive histone marks, consequently fostering a more transcriptionally active chromatin state. KDM1A is a flavin-dependent demethylase that removes methyl groups from H3K4me1/2 (activating mark) and H3K9me1/2 (repressive mark) in a context-dependent manner. The PFOS-induced upregulation of KDM1A (**Figure 8B**) results in the loss of H3K9me2 repression, permitting the transcriptional activation of oncogenic pathways, altering differentiation programs, promoting stemness-like characteristics in cancer cells, and enhancing proliferation and epithelial–mesenchymal transition (EMT), which are key attributes of tumour progression. KDM4C, a JmjC-domain-containing histone demethylase, specifically demethylates H3K9me3/me2 (marks of heterochromatin), prompting chromatin decondensation and transcriptional activation of oncogenes, which contributes to increased genomic instability as repetitive DNA elements lose heterochromatic repression and increase the expression of MYC and other proliferation-associated genes, invigorating tumour growth. KDM4C has also been shown to promote the activation of HIF1α/VEGFA signaling through the costimulatory factor STAT3 in NSCLC [141]. Thus, the overexpression of KDM1A and KDM4C triggered by PFOS creates a chromatin environment favoring carcinogenesis by promoting oncogenic transcription, diminishing cellular differentiation, and enabling metastatic progression [129]. The concurrent loss of DNA and histone methylation leads to a significant transformation in the epigenetic landscape of cells subjected to PFOS exposure. Ordinarily, DNA methylation and H3K9me3 collaborate to establish and maintain heterochromatin. However, PFOS undermines both marks, resulting in a chromatin state that is more permissive to transcription, thus facilitating oncogenic activation and cell transformation. This observation indicates that PFOS functions as an epigenetic disruptor, potentially increasing the risk of cancer, metabolic disorders, and developmental abnormalities.

Another study employing advanced super-resolution imaging techniques and machine-learning tools elucidated that PFOS can alter the spatial configuration of repressive heterochromatin marks, namely H3-lysine-9-trimethylation (H3K9me3) and H3-lysine-27-trimethylation (H3K27me3), within renal carcinoma cells [142]. Exposure to PFOS does not appear to significantly impact the overall quantities of H3K9me3 or H3K27me3. Instead, it leads to the dispersion and de-clustering of these histone marks. H3K9me3 and H3K27me3 are typically densely clustered in heterochromatic regions, particularly adjacent to the nuclear periphery, to maintain gene silencing. PFOS disrupts this structural organization and renders the repressive domains more diffuse, possibly contributing to aberrant gene expression. Their studies also revealed that PFOS is linked with an increased expression of KDM4A. KDM4A is a histone demethylase responsible for removing methyl groups from H3K9me3 [143]. This enzymatic activity can potentially diminish local concentrations of H3K9me3, thereby weakening chromatin compaction and facilitating a more open euchromatic structure. Although KDM4A does not directly demethylate H3K27me3, the overarching imbalance in chromatin-modifying enzymes may indirectly affect the dynamics of H3K27me3 [142]. These findings suggest that PFOS functions as an epigenetic disruptor, interfering with chromatin modifiers, transcriptional repressors, and nuclear scaffolding proteins. Consequently, this interference may relocate repressive histone marks, undermining their efficacy in gene silencing and promoting the activation of stress, inflammation, or proliferation genes. PFOS did not significantly decrease the expression level of histone demethylase KMT1A [142]. KMT1A (SUV39H1) is a histone methyltransferase (HMT) that catalyses explicitly the trimethylation of histone H3 at lysine 9 (H3K9me3), a characteristic marker of transcriptional repression [144,145,146]. By establishing and preserving heterochromatin, KMT1A is indispensable for genomic stability, gene silencing, and cellular differentiation. KMT1A operates as a chromatin modifier, engaging with heterochromatin protein 1 (HP1) to compact chromatin and inhibit transcription. This repression is crucial for silencing repetitive DNA sequences, maintaining X-chromosome inactivation, and regulating developmental genes [147]. Furthermore, KMT1A collaborates with DNA methyltransferases (DNMTs) and histone deacetylases (HDACs) to reinforce stable gene silencing throughout cellular generations. These alterations, perhaps, are potential drivers of toxicity induced by PFOS.

### 3.6. Endocrine Disruption

PFOS has been recognized as an endocrine disruptor that may potentially exert adverse effects on multiple hormonal functions, including thyroid health [148]. Previous research has highlighted concerns regarding the impact of PFOS on the thyroid gland, although findings have been inconsistent across in vitro studies and different study populations. Thyroid hormones (TH) are crucial in numerous biochemical processes, such as regulating energy expenditure, growth, and neurodevelopment from fetal development through infancy. Additionally, these hormones continue to influence metabolic processes throughout adulthood [149]. Disruptions to thyroid function caused by PFOS exposure could, therefore, have significant long-term health implications [150]. Thyroid hormones also play a vital role in brain formation during fetal development, guiding key processes such as neurogenesis, neuronal migration, synapse formation, and myelination. Insufficient thyroid hormone levels during critical periods of neurodevelopment can lead to long-term intellectual and behavioural impairments [150]. Beyond early development, thyroid hormones regulate metabolism throughout infancy and adulthood by acting on various organs, including the brain, white and brown adipose tissue, skeletal muscle, liver, and pancreas. Their secretion is controlled by the hypothalamic-pituitary-thyroid (HPT) axis. The hypothalamus initiates thyroid hormone regulation by releasing thyrotropin-releasing hormone (TRH) into the pituitary portal system. This stimulates the secretion of thyroid-stimulating hormone (TSH) by the anterior pituitary. TSH then stimulates the thyroid gland to produce and release thyroid hormones, mainly thyroxine (T4) and triiodothyronine (T3). While the thyroid gland mainly secretes T4, about 80% of circulating T3 is generated through the enzymatic conversion of T4 in peripheral tissues, including the brain. This conversion is facilitated by three types of deiodinase enzymes, which are essential for modulating thyroid hormone activity by controlling the local concentration of active T3 at various biological levels [151]. Excess thyroid hormones, a condition known as hyperthyroidism or thyrotoxicosis, result in a hypermetabolic state characterized by increased basal energy expenditure, weight loss, enhanced fat breakdown, and elevated glucose production [152]. In contrast, insufficient thyroid hormone levels lead to hypothyroidism, a hypometabolic state marked by reduced energy expenditure, weight gain, decreased lipolysis, and lower gluconeogenesis [150].

**Figure 9:**
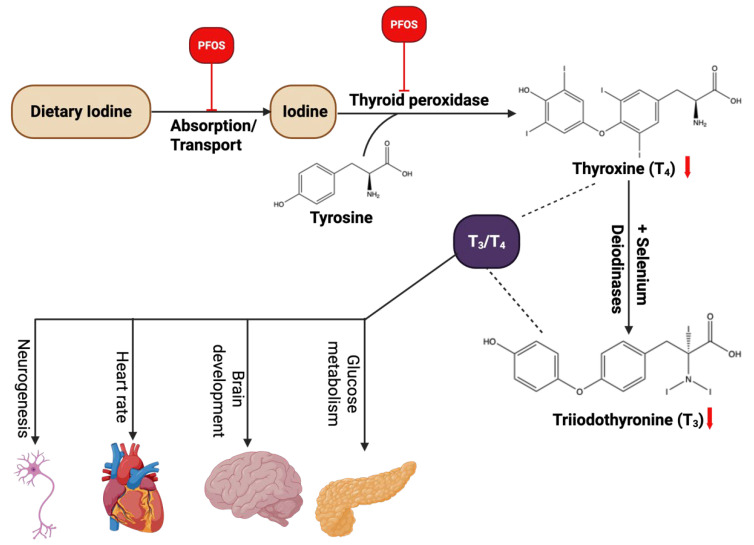
PFOS-mediated endocrine function disruption. PFOS inhibits iodine uptake, suppresses thyroid peroxidase activity, and disrupts the conversion of thyroxine (T4) to triiodothyronine (T3) by deiodinases. These disruptions lead to reduced levels of T4 and T3, impairing key physiological processes including neurogenesis, brain development, heart rate regulation, and glucose metabolism. Generated with BioRender (https://app.biorender.com, accessed on 15 May 2025).

Recent literature has examined how PFOS disrupts thyroid function, offering insights into the molecular pathways involved in this endocrine interference [153,154,155,156,157]. PFOS exposure can disrupt the biosynthesis and secretion of thyroid hormones at multiple stages (**Figure 9**). Several mechanisms have been proposed, including impairment of iodine uptake, in which PFOS may compete with iodine or directly inhibit the sodium/iodide symporter (NIS), thus reducing the availability of iodine for thyroid hormone production [155]; interference with thyroglobulin synthesis, wherein PFOS could disrupt the formation of thyroglobulin, a critical precursor in thyroid hormone production [156]; modification of thyroperoxidase (TPO) activity, through which PFOS may alter the activity of TPO, an enzyme essential for the synthesis of thyroid hormones; and disruption of thyroid hormone regulation, in which PFOS could interfere with feedback mechanisms, affect deiodinase enzyme activity, or alter thyroid hormone binding proteins, thereby disrupting the signaling and biological effects of thyroid hormones [157]. These disruptions underscore the potential endocrine-disrupting properties of PFOS and their consequential impacts on thyroid function. According to the Endocrine Society, endocrine-disrupting chemicals (EDCs) are exogenous chemicals or mixtures of chemicals that interfere with any part of hormone action. Considering the endocrine system’s crucial role in controlling various biological and physiological processes, any disruption can lead to the development of several diseases [158].

The thyroid gland and its hormones are essential for maintaining metabolic homeostasis and ensuring proper development during the fetal and childhood stages. Disruptions in thyroid function can lead to significant health issues. Several studies have identified PFOS as an endocrine-disrupting compound capable of interfering with hormonal balance and impairing normal endocrine system activity [149,157]. Experimental research using cultured thyroid cells has shown that PFOS exposure in vitro can lead to thyroid dysfunction [149,157]. PFOS has been observed to accumulate within thyroid cells, and at elevated concentrations, it can exert cytotoxic effects [159]. Moreover, PFOS has been shown to suppress thyroperoxidase (TPO) activity in thyroid tumor cell lines, potentially disrupting the synthesis of thyroid hormones. It may also function as a thyroid hormone receptor (THR) agonist, as demonstrated by luciferase reporter assays involving THRα and THRβ in GH3 cells [160]. Conti et al. [155] reported that PFOS can inhibit iodide uptake in FRTL-5 thyroid cells by interfering with the sodium/iodide symporter (NIS). This inhibitory effect was also observed in non-thyroid cells engineered to express NIS, further supporting the potential of PFAS to impair iodine transport and thyroid function. An in vitro study by Croce et al. [161] evaluated the thyroid-disrupting potential of both long-chain PFAS (such as PFOS and PFOA) and short-chain variants, including PFBS, PFBA, PFPA, and PFPeA. The results showed that while long-chain PFAS caused cytotoxicity and disrupted TSH-dependent cAMP production in rat thyroid cells, short-chain PFAS did not exhibit these effects. This suggests that the impact of PFAS on thyroid function may depend on their molecular structure and chain length. Collectively, these findings indicate that PFOS exposure can impair endocrine function in humans, potentially affecting neurodevelopment, cardiovascular regulation, brain maturation, and glucose metabolism.

### 3.7. Immunosuppressive Effects and Inflammatory Responses of PFOS

An individual’s health depends on maintaining homeostasis within the immune system. When appropriately balanced, the immune system is capable of recognizing threats, initiating an appropriate response, repairing tissue damage incurred, and subsequently returning to a resting state [162]. Given that the immune system is dispersed across various tissues and organ systems, it is highly vulnerable to toxic substances through multiple exposure pathways. Considering its complex composition of diverse cell types with specialized functions, exposure to toxicants can impair immune function in various ways, potentially leading to immunosuppression, excessive immune activation, or both. Furthermore, disruptions to immune system development, such as those caused by exposure to immunotoxins, tend to yield more severe and enduring consequences than similar disturbances occurring in adulthood. An increasing body of research evidence indicates that PFOS is a significant immunotoxin, thereby raising concerns regarding its impact on immune health [163,164,165]. The most compelling epidemiological evidence of PFOS-related immunotoxicity is the reduction in antibody production following vaccination, especially in children receiving tetanus and diphtheria vaccines. Antibodies play a crucial role in the adaptive immune system, helping to combat infections and mitigate damage from harmful agents. Specifically, Grandjean et al. [166] reported that PFOS negatively impacted the antibody concentrations of study participants of 5 years of age. As PFOS concentrations rose, exposure was linked to a 39% drop in diphtheria antibody levels. A subsequent study involving the same cohort at age 13 confirmed that elevated PFOS levels were associated with reduced antibody levels, indicating a lasting immunotoxic effect [167]. Zhang et al. [168] analyzed multiple epidemiological studies in a systematic review and meta-analysis. They found that exposure to PFOS is linked to lower tetanus antibody levels in children, backing up evidence of immunotoxicity. Animal studies also support the idea that exposure to PFOS can reduce the body’s ability to produce specific antibodies [169].

**Figure 10:**
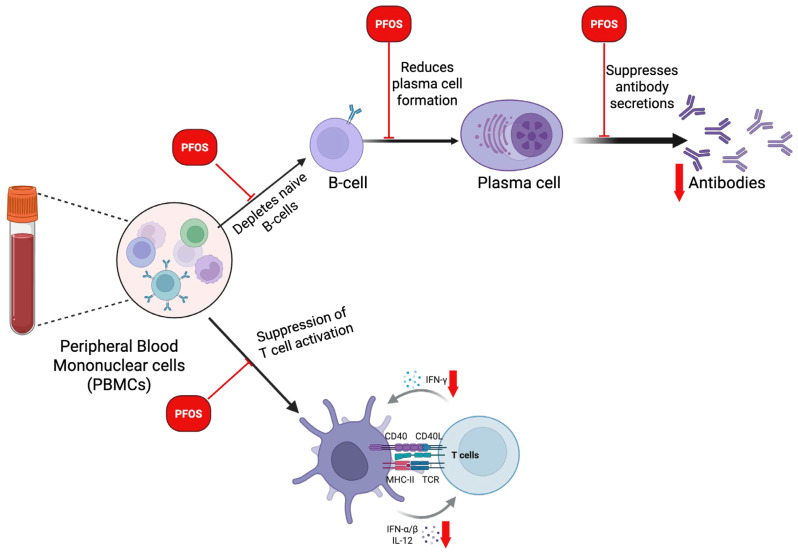
PFOS-mediated disruptions of B-cell and T-cell function. PFOS impairs adaptive immune responses by targeting key cellular processes. In B-cells, PFOS depletes naïve B-cell populations, reduces plasma cell differentiation, and suppresses antibody production. In T-cells, PFOS inhibits activation markers (CD3, CD4/CD8, MHC-TCR) and cytokine signaling (e.g., IL-2, IFN-γ), leading to diminished T-cell activation and proliferation. These disruptions collectively contribute to immunosuppression. Generated with BioRender (https://app.biorender.com, accessed on 15 May 2025).

Additionally, research has linked PFOS exposure to an increased risk of respiratory and gastrointestinal infections, particularly in children exposed to the uterus. These findings and recent studies emphasize the immunosuppressive effects of PFOS [170]. These findings reveal that exposure to PFOS, particularly in neonates and children, may impair B-cell function by depleting naïve B cells, disrupting B-cell signaling needed for B-cell differentiation, reducing plasma cell formation, and suppressing the biosynthesis of antibodies essential for mounting an effective humoral immune response against foreign pathogens (**Figure 10**). An increasing body of evidence indicates that exposure to PFOS has notable immunotoxic effects on T cell populations, resulting in disruptions to their development and functional capacity. Research on animal models has consistently shown that PFOS exposure decreases total T-cell counts, particularly impacting CD4^+^helper T cells and CD8^+^cytotoxic T cells. This reduction can be partially attributed to thymic atrophy, as PFOS induces the shrinkage of the thymus, thereby impairing the maturation and output of naïve T cells [171]. Furthermore, beyond mere numerical reductions, PFOS modifies the distributions of T-cell subsets. Such exposure has been linked to a decreased proportion of naïve T cells and an increased prevalence of memory T cells within peripheral lymphoid organs [172]. These shifts may jeopardize the immune system’s capacity to respond effectively to novel antigens.

Additionally, functional impairment of T cells constitutes a notable characteristic of PFOS immunotoxicity. PFOS diminishes the expression of critical activation markers, such as CD69 and CD25, which culminates in reduced T-cell activation following antigenic stimulation [173]. Moreover, PFOS inhibits T-cell proliferation, an essential process for expanding antigen-specific clones during immune responses. The apoptosis of T cells is another mechanism through which PFOS undermines immunity. Elevated rates of apoptosis in T-cells have been documented following PFOS exposure, further diminishing the available pool of functional lymphocytes [174]. At the cytokine level, PFOS disrupts the secretion of crucial signaling molecules such as interleukin-2 (IL-2) and interferon-gamma (IFN-γ), which are vital for T-cell growth, survival, and effector function [175]. This dysregulation of cytokines impairs communication between immune cells and reduces the overall efficacy of immune responses. It is important to note that PFOS alters the equilibrium between T-helper 1 (Th1) and T-helper 2 (Th2) responses. There is a noted suppression of Th1-mediated immunity, characterized by reduced IFN-γ production, alongside a relative promotion of Th2 responses, which may render the host susceptible to allergic conditions and diminish cellular immunity essential for combating viral and intracellular bacterial infections [172]. These findings illustrate that PFOS profoundly disrupts T-cell biology at multiple levels, affecting their development, survival, activation, and cytokine signaling pathways (**Figure 10**), undermining the host’s adaptive immune defense. Given the central role of T cells in orchestrating immune responses, the dysfunction induced by PFOS in T cells signifies a critical mechanism contributing to the broader immunosuppressive effects observed in exposed populations. Furthermore, PFOS has been found to suppress immune activity in trophoblast cells. Szilagyi et al. [176] found that treatment with PFOS led to a reduction in the expression of chemokines (e.g., CCL2), chemokine receptors (e.g., CCR4), and inflammatory enzymes (e.g., ALOX15) in trophoblasts, which are involved in migration. These inflammatory proteins play a crucial role in establishing optimal blood flow between the placenta and the maternal endometrium.

## 4. PFOS and Carcinogenesis

### 4.1. PFOS and the Hallmarks of Carcinogenesis

The Hanahan and Weinberg model describes six fundamental traits referred to as the hallmarks of cancer [177]. The first hallmark discussed is how normal cells require growth factors to divide, but cancer cells find ways to grow without external stimulation. They may produce their own growth factors, overexpress receptors for these signals, or activate signaling pathways downstream of these receptors, such as the Ras-MAPK or PI3K-Akt pathways. This allows them to maintain constant division, even in the absence of normal regulatory cues. For example, mutations in the EGFR or HER2 receptors can cause continuous activation of growth signaling, leading to uncontrolled proliferation [178]. The second hallmark is evading growth suppressors. Healthy cells have tumor suppressor genes, such as p53 and Rb, that act as brakes to prevent unchecked division. Cancer cells bypass these mechanisms by mutating or inactivating these genes. The loss of p53, for instance, removes a key checkpoint that would normally trigger cell-cycle arrest or apoptosis in response to DNA damage. Similarly, inactivation of Rb allows cells to enter the cell cycle even when growth conditions are unfavorable [179]. The third hallmark is resisting cell death, also known as evading apoptosis. Normal cells undergo programmed cell death when they are damaged or no longer needed. Cancer cells, however, develop ways to resist this fate. They often overexpress anti-apoptotic proteins such as Bcl-2 or downregulate pro-apoptotic proteins like Bax. Some also interfere with death receptor signaling (for example, the Fas/FasL pathway), ensuring their survival under conditions that would normally trigger death in normal cells [180]. The fourth hallmark, enabling replicative immortality, refers to the ability of cancer cells to divide indefinitely. Normal cells can only divide a limited number of times because their telomeres, protective caps at the ends of chromosomes, shorten with each division. Cancer cells counter this by reactivating telomerase, the enzyme that rebuilds telomeres, allowing them to escape the natural limit on cellular lifespan and achieve immortality [181]. The fifth hallmark, inducing angiogenesis, describes how tumors stimulate the formation of new blood vessels. As tumors grow beyond a small size, they require their own blood supply to obtain oxygen and nutrients. Cancer cells release signaling molecules such as vascular endothelial growth factor (VEGF) to recruit new blood vessels. These vessels are often irregular and leaky but support the tumor’s continued growth and metastasis [182]. The sixth hallmark, activating invasion and metastasis, explains how cancer spreads from its site of origin to distant organs. Tumor cells acquire the ability to detach from their original tissue, degrade surrounding extracellular matrix using enzymes such as matrix metalloproteinases (MMPs), and migrate through the bloodstream or lymphatic system. A process known as epithelial–mesenchymal transition (EMT) often facilitates this change, giving cancer cells increased mobility and invasiveness [183].

Beyond these original six, Hanahan and Weinberg introduced two new hallmarks that had emerged from research in the previous decades. The first of these is reprogramming energy metabolism. Normal cells primarily use oxidative phosphorylation to produce energy, but cancer cells often rely on glycolysis, even in the presence of oxygen, a phenomenon known as the Warburg effect. This metabolic reprogramming supports rapid cell growth by providing building blocks for biosynthesis rather than just energy. Cancer cells also alter mitochondrial function and nutrient uptake to suit their needs [184]. The second newly added hallmark is evading immune destruction. The immune system can detect and eliminate emerging cancer cells, but tumors evolve strategies to avoid immune surveillance. They may express proteins like PDL1, which bind to immune checkpoints (such as PD-1 on T cells) and inhibit immune attack. They can also recruit immunosuppressive cells like regulatory T cells (Tregs) and myeloid-derived suppressor cells (MDSCs), which weaken the body’s anti-tumor response. This hallmark laid the conceptual foundation for the development of immune checkpoint inhibitors, a major breakthrough in cancer therapy [185]. In addition to these eight hallmarks, the authors identified two enabling characteristics that make it easier for tumors to acquire these malignant traits. The first is genome instability and mutation, which refers to the tendency of cancer cells to accumulate genetic changes. Defects in DNA repair mechanisms, such as mutations in BRCA1 or mismatch repair genes, lead to an increased mutation rate, providing the raw material for evolution within the tumor. The second enabling characteristic is tumor-promoting inflammation. Chronic inflammation in the tumor microenvironment can supply growth factors, survival signals, and pro-angiogenic factors that accelerate tumor progression. Cytokines such as TNF-α and IL-6, produced by immune cells, create a feedback loop that sustains proliferation and metastasis. Hanahan and Weinberg [177] also emphasized the role of the tumor microenvironment (TME), a complex ecosystem consisting of non-cancerous cells, including immune cells, fibroblasts, and blood vessels, that interact with cancer cells. They argued that cancer is not just a cell-autonomous disease but a dynamic ecosystem where tumor and stromal cells co-evolve. The microenvironment provides essential support for growth, angiogenesis, immune evasion, and metastasis [186]. PFOS may act as a multifaceted carcinogen by enhancing several hallmarks of cancer, thereby promoting tumor initiation, progression, and resistance to therapy. Studies have shown that PFOS induces carcinogenesis through multiple mechanisms that align with the hallmarks of cancer, including sustaining proliferative signaling through the activation of PI3K/Akt and MAPK pathways, interfering with p53 signaling, evading apoptosis, orchestrating angiogenesis, reprogramming energy metabolism, suppressing immune function, inducing genomic instability and promoting inflammation in tumors [42,172,187,188].

In 2012, at a workshop organized by the International Agency for Research on Cancer (IARC), participants noted that human carcinogens share one or more characteristics that lead to cancer. This observation led to the development of 10 key characteristics (KCs) of human carcinogens [189]. These 10 key characteristics provide a systematic framework for understanding how chemicals such as PFOS can orchestrate cancer. Instead of focusing on individual mechanisms, the KCs describe common biological hallmarks shared by known human carcinogens. These hallmarks include the ability to damage DNA directly or indirectly, interfere with the mechanisms of DNA repair, alter gene expression through epigenetic changes, induce oxidative stress and inflammation, suppress immune function and disrupt key cellular signaling pathways. Carcinogens may also promote cellular immortality, enhance abnormal cell proliferation, or alter the cellular microenvironment to support tumor growth. This framework helps scientists organize mechanistic data from toxicological, molecular, and epidemiological studies, making it easier to identify potential carcinogens [120]. Experimental evidence suggests that PFOS exhibits several of these characteristics. Although PFOS is not inherently electrophilic and does not undergo metabolic activation to electrophile intermediates, it can indirectly induce cellular damage through redox imbalance. Experimental and in vivo studies have shown that PFOS exposure leads to oxidative stress, which is characterized by increased ROS, decreased glutathione (GSH), and inhibition of antioxidant enzymes such as catalase (CAT) and superoxide dismutase (SOD) [190]. PFOS exposure triggers chronic inflammation, evidenced by elevated IL-6, TNF-α, and IL-1β expression and activation of NF-kB signaling. It also exhibits immunosuppressive effects, reducing antibody response, lymphocyte proliferation, and macrophage activity, which may impair the immune system and promote tumor progression [173]. PFOS interacts with several nuclear receptors, including PPARα, and interferes with estrogen and androgen receptor signaling, thereby modulating lipid metabolism, hormone balance, and cell proliferation [191]. Moreover, PFOS disturbs apoptosis and enhances survival signaling via PI3K/Akt and MAPK pathways, promoting abnormal cell proliferation and resistance to cell death. Collectively, while PFOS does not comply with all IARC KCs, it demonstrates strong alignment with those involving oxidative stress, inflammation, epigenetic alteration, immune suppression, receptor modulation, and dysregulation of cell proliferation and apoptosis. These overlapping pathways suggest that PFOS may promote carcinogenesis through an indirect mechanism of tumor initiation and progression rather than direct genotoxicity.

### 4.2. Prostate Cancer: Mechanism and Carcinogenesis

PCa is the most prevalent solid cancers that occur in men across Western countries. Globally, PCa ranks as the fourth most common malignancy in men, though its incidence varies across different regions and ethnic groups [192,193]. The lowest rates have been recorded in Asia, such as Tianjin, China, with 1.9 cases per 100,000 men yearly. In contrast, the highest incidence is observed in North America and Scandinavia, particularly among African American men, reaching up to 272 cases per 100,000 annually [194]. The development of PCa is a multi-step process that involves both genetic and biochemical changes. The first stage, “initiation,” involves a genetic alteration that grants the affected cell malignant potential. This is followed by “promotion,” where additional irreversible genetic changes drive abnormal cell growth, leading to tumor progression. Various factors contribute to these changes, including genetic predisposition and environmental influences. There is a crucial interplay between inherited genetic factors and external ecological exposures in the onset and progression of PCa. The prostate is the primary accessory gland in the male reproductive system, playing a vital role in reproductive function. Its epithelial cells are known to produce prostatic fluid, which contains key molecules that support not only the gland’s activity but also crucial reproductive processes, including ejaculation, sperm motility, and capacitation factors essential for male fertility [195,196]. Key components of prostatic fluid include proteins from the kallikrein family, notably prostate-specific antigen (PSA), as well as vital elements such as zinc and intermediates of the Krebs cycle [195,196]. PSA, a cysteine protease synthesized by the epithelial cells of the prostate and secreted into the ductal lumen, plays a crucial role by degrading semenogelins I and II, the proteins responsible for forming the gel-like structure of semen. PSA is subsequently expelled during ejaculation [195,196]. The Reduction in PSA levels may impair the sperm’s ability to fertilize, while elevated PSA levels are commonly utilized as a diagnostic biomarker for PCa [195,196].

PCa exhibits significant clinical variability, ranging from aggressive forms that progress to metastasis to slow-growing, asymptomatic cases with a more indolent course [197,198]. However, the cellular and biological mechanisms driving its high prevalence remain poorly understood. Standard treatment strategies primarily include surgical intervention and radiotherapy. For patients who are not eligible for these options, androgen deprivation therapy is commonly employed as an alternative [199]. Androgen receptor (AR) signaling plays an important role in the transformation of normal prostate epithelial cells into PCa cells. These Androgenic hormones are essential for the normal growth and function of the prostate, acting through the androgen receptor (AR). AR is a ligand-activated transcription factor that belongs to the nuclear receptor superfamily. Specifically, it is classified as NR3C4 (nuclear receptor subfamily 3, group C, member 4) [200].

**Figure 11:**
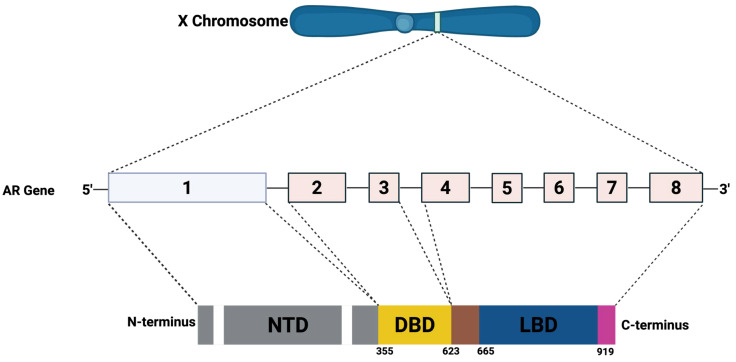
Structure of the androgen receptor (AR) gene. The AR gene is located on the X chromosome and comprises eight exons. The encoded protein includes three major functional domains: the N-terminal domain (NTD; residues 1–555), DNA-binding domain (DBD; residues 556–623), and ligand-binding domain (LBD; residues 666–919). These domains are essential for AR-mediated transcriptional regulation and play critical roles in PCa development and progression. Generated with BioRender (https://app.biorender.com, accessed on 10 June 2025).

The AR gene is situated on the X chromosome at location Xq11–12 (**Figure 11**). It comprises 8 exons interspersed with introns that vary in length from approximately 0.7 to 2.6 kilobases. This gene encodes a protein of about 920 amino acids, sharing structural features with other members of the nuclear receptor family. The AR protein is organized into 4 principal functional regions: a flexible N-terminal domain (NTD; residues 1–558), a DNA-binding domain (DBD; residues 558–624), a C-terminal domain (residues 676–919), and a ligand-binding domain (LBD).

**Figure 12:**
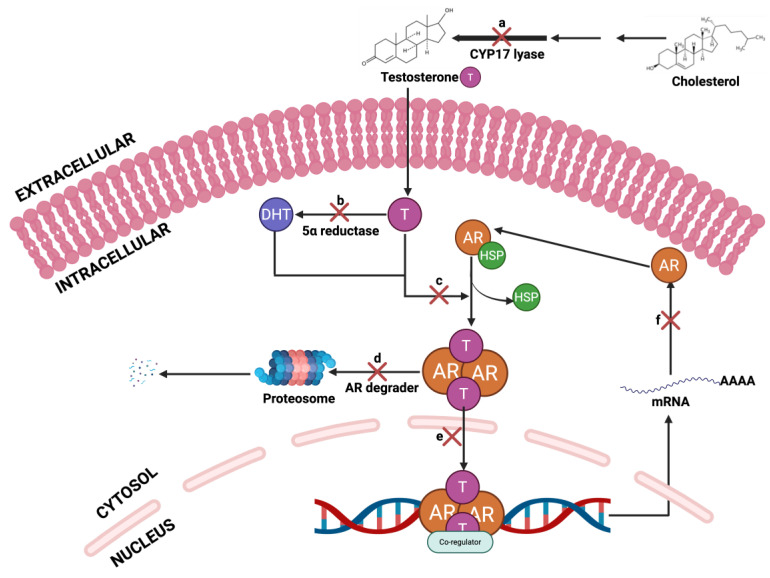
AR signaling pathway in PCa. The diagram illustrates the molecular cascade beginning with cholesterol conversion to testosterone via CYP17 lyase. Testosterone is further metabolized to dihydrotestosterone (DHT) by 5α-reductase, which binds to cytosolic AR, triggering dissociation from heat shock proteins (HSPs) and enabling nuclear translocation. Inside the nucleus, the AR-DHT complex binds to androgen response elements (AREs) on DNA, initiating transcription of genes that drive PCa progression. Points of therapeutic inhibition are highlighted: (a) CYP17 lyase inhibitors (e.g., abiraterone) block androgen biosynthesis. (b) Androgen deprivation therapy reduces circulating testosterone. (c) 5α-reductase inhibitors (e.g., finasteride, dutasteride) prevent DHT formation. (d) AR inhibition or degradation targets the androgen receptor directly, either by blocking ligand binding, preventing nuclear translocation, or promoting proteasomal degradation. Degraders such as PROTAC act at this level. (e) AR antagonists (e.g., enzalutamide) inhibit receptor activation or promote degradation. (f) Transcriptional inhibitors block AR-mediated gene expression. Generated with BioRender (https://app.biorender.com, accessed on 10 June 2025).

The AR signaling pathway (**Figure 12**) is a central driver of PCa progression, and therapeutic interventions have been developed to inhibit this axis at multiple molecular checkpoints. The pathway initiates with the biosynthesis of testosterone from cholesterol, catalyzed by CYP17 lyase; inhibition at this step using agents such as abiraterone effectively reduces systemic androgen levels. Testosterone is subsequently converted to the more potent dihydrotestosterone (DHT) via 5α-reductase, a process blocked by inhibitors like finasteride and dutasteride. Upon ligand binding, AR undergoes conformational activation and forms a cytoplasmic complex that can be inhibited by anti-androgens such as enzalutamide, bicalutamide, Seviteronel, TRC253, CR1447, flutamide and darolutamide [201,202,203], which competitively block androgen binding and prevent AR activation. Additionally, AR degraders promote proteasomal degradation of the receptor, further disrupting AR signaling [204,205]. AR degraders, including proteolysis-targeting chimaeras (PROTACs), selective AR degraders (SARDs), and hydrophobic tags (HyT), have been developed to target and treat metastatic PCa [206]. Among AR degraders, PROTACs represent a novel and highly promising therapeutic strategy. PROTACs are bifunctional molecules that simultaneously bind to the AR and an E3 ubiquitin ligase, facilitating the ubiquitination and subsequent proteasomal degradation of the AR protein. Unlike conventional AR antagonists that merely block receptor activity, PROTACs eliminate the receptor entirely, thereby overcoming resistance mechanisms such as AR overexpression, mutations, and splice variants. This targeted degradation approach offers a more sustained suppression of AR signaling and has shown efficacy in preclinical models of castration-resistant PCa (CRPCa). Several AR-targeting degraders are currently under investigation, with early candidates demonstrating potent anti-tumor activity and favorable pharmacokinetic profiles [206,207,208]. The activated AR complex normally translocates into the nucleus and binds to androgen response elements (AREs) on DNA to initiate transcription; this step is also targeted by agents that inhibit nuclear translocation or DNA binding. Finally, AR-mediated transcription of target genes drives tumor growth and survival, and transcriptional inhibitors aim to suppress this terminal output of AR signaling. Collectively, these points of inhibition represent a comprehensive strategy to attenuate AR activity and mitigate PCa progression.

AR blockade, otherwise known as ADT (anti-androgen therapy), has been demonstrated to induce tumor regression in up to 80% of PCa patients [209]. However, over time, PCa often resumes growth despite continued ADT [209]. Resistance to ADT may be caused by several mechanisms linked to androgen signaling, including androgen production within the tumor itself or by the adrenal glands, AR gene amplification or overexpression, the presence of AR mutations, and the development of constitutively active AR splice variants [210].

#### Evidence of PFOS in Prostate Cancer Development

Wen et al. [211] presented the first direct evidence that PFOS exerts unambiguous biological effects on the human prostate stem/progenitor cell (SPC) population, potentially contributing to an increased carcinogenic risk and tumor progression within the prostate gland. The study utilized a prostasphere model derived from healthy human prostate epithelial cells. The results showed that chronic exposure to PFOS at environmentally relevant concentrations led to a consistent increase in spheroid formation across serial passages, even when the same number of cells was seeded each time. Given that prostaspheres under these culture conditions are predominantly initiated by resident stem cells [212], this sustained increase strongly indicates enhanced symmetric self-renewal of prostate SPCs in the presence of PFOS, facilitating stem cell pool expansion. This is especially significant, considering the established link between cancer risk and the frequency of normal stem cell divisions in various tissues, including the prostate [213,214]. Moreover, PFOS exposure promoted the formation of larger spheroids, indicative of increased proliferation among progenitor cells, which constitute the majority population in prostaspheres [212]. Previous studies have shown that other endocrine-disrupting chemicals (EDCs), such as bisphenol A (BPA), dioxins, and inorganic arsenic, also target prostate SPCs, probably because they express various steroid hormone receptors [215].

The study conducted by Alyssa et al. [216], observed a positive association between PFOS and PCa in men aged ≥70 years. Their finding suggests that older men may be more susceptible to PCa owing to reduced immune function. Also, men with the highest PFOS exposure had about an 18% higher risk of developing PCa compared to those with the lowest exposure. However, when PFOS levels were analyzed as a continuous variable, no clear association with PCa risk was found. But when examined using a non-parametric model, the risk of PCa seemed to increase with PFOS levels up to a certain point (log_2_ PFOS = 4.2), possibly explaining the null result in the continuous model. Their findings suggest a possible but uncertain link between PFOS exposure and PCa risk; the observed higher risk at high PFOS levels could be real or just due to chance. Previous studies in the general population have also reported no clear or consistent association between PFOS and PCa [216].

Similarly, the study by Wen et al. [211], observed a strong expression of PPARα and RXRα was detected within prostate SPCs, an important finding given that many biological actions of PFOS are mediated through PPARα and its heterodimeric partner RXR [217,218]. These receptor-mediated interactions may initiate signaling cascades that influence SPC behavior and phenotype. Two primary downstream mechanisms were explored: transcriptomic and metabolic alterations induced by PFAS exposure. Single-cell RNA sequencing (scRNA-seq) revealed the emergence of a unique progenitor cell cluster in PFOS-treated spheroids that was absent in controls. Leveraging previously established KRT-based lineage markers [219], the new cluster was identified as an aberrant early-stage luminal progenitor population, a cell type increasingly implicated in prostate carcinogenesis [220]. Transcriptome-wide analysis indicated an upregulation of gene pathways linked to cell proliferation (G2M checkpoint, E2F targets, mitotic spindle) and oncogenic signaling (KRAS, MYC, TNFα via NF-κB, IL-6–JAK–STAT3, and TGF-β). The enrichment of these pathways corresponds with previous findings that associate therapeutic-resistant metastatic PCa with stemness-related signatures, including inflammatory and cytokine-driven signaling [221]. Since PPARs play a central role in metabolic regulation, they also profiled metabolic alterations in SPCs exposed to PFOS. Metabolomic analyses demonstrated elevated glycine and serine biosynthesis and evidence of enhanced aerobic glycolysis (Warburg effect), particularly in PFOS-treated samples.

Metabolic regulation is increasingly recognized as a critical factor in stem cell fate decisions and tumorigenesis [222,223]. Like pluripotent stem cells, cancer stem cells display high glucose uptake and lactate production, known as the Warburg effect [224,225]. Enhanced glycolysis and lactate secretion following PFOS exposure may therefore drive both self-renewal and impaired differentiation. Additionally, recent studies in epidermal pre-malignant stem cells have underscored the importance of serine biosynthesis from glucose as a rate-limiting step in stem cell proliferation, a metabolic phenotype mirrored in PCa stem-like cells [225]. Their Finding of pronounced upregulation of serine and glycine metabolism, particularly following long-term PFOS exposure, raises the possibility that PFOS may promote a pre-malignant state by fueling key biosynthetic pathways. To determine whether these effects translate into enhanced tumorigenic potential, they utilized a xenograft model with RWPE-2 prostate epithelial cells (harboring k-RAS activation) implanted into nude mice. PFOS administration significantly accelerated tumor growth over 40 days. Given that RWPE-2 cells contain a subpopulation with cancer stem-like properties, this finding suggests that PFOS may act not only on differentiated tumor cells but also on the cancer stem cell compartment, further promoting tumor expansion [226].

These findings (Table 1) provide compelling mechanistic evidence that PFOS directly impacts human prostate stem and progenitor cells by promoting self-renewal, increasing proliferative capacity, and disrupting normal differentiation trajectories. These effects are mediated, at least in part, through PFOS-induced alterations in transcriptional and metabolic programs, pushing SPCs toward a pre-malignant phenotype. Furthermore, in vivo evidence from xenograft models supports the role of PFOS in enhancing tumor growth, likely via modulation of the cancer stem cell niche. These mechanistic insights offer a plausible biological framework for the observed epidemiological associations between PFOS exposure and increased PCa risk and underscore the need for further investigation into the long-term health effects of chronic PFOS exposure.

### 4.3. Breast Cancer: Mechanism and Carcinogenesis

BCa remains one of the most commonly diagnosed malignancies among women globally [192,193,227,228]. The classification of BCa subtypes is primarily based on hormone receptor expression. The first group includes tumors that are positive for estrogen receptor (ER) and/or progesterone receptor (PR). The second group consists of tumors that overexpress human epidermal growth factor receptor 2 (HER2), regardless of ER or PR status. The third group, known as triple-negative breast cancer (TNBC), lacks expression of ER, PR, and HER2 [229]. Advances in gene expression profiling using microarray technologies have further refined BCa classification into intrinsic molecular subtypes. These include: Luminal A, characterized by ER+/PR+ status, high expression of luminal-related genes (e.g., ESR1, GATA3, XBP1, FOXA1), and low proliferation indicated by low Ki-67 levels; Luminal B, which also expresses ER but shows lower levels of luminal genes (e.g., PGR, FOXA1) and higher Ki-67 (>20%); HER2-enriched, defined by HER2 positivity and moderate luminal gene expression, though not all HER2+ tumors fall into this category; Basal-like, which exhibits elevated expression of EGFR and basal cytokeratins with minimal luminal gene expression; and Claudin-low, a subtype that is negative for ER, PR, and HER2, and also shows reduced expression of claudin 3/4/7 and E-cadherin [230].

ERs are key regulators in the development and progression of several hormone-related cancers, including those of the breast, endometrium, and ovaries [231,232,233]. Estrogens, which are small, lipophilic steroid hormones, exist in four main forms: estrone (E1), estradiol (E2), estriol (E3), and estetrol (E4). Among these, E2 is the most biologically active and is often referred to simply as estrogen. E4, produced exclusively during pregnancy in the fetal liver, exhibits tissue-specific effects [234]. Structurally, estrogens share a steroidal framework composed of four fused rings, with the phenolic A-ring playing a crucial role in receptor binding. Like other steroid hormones, estrogens are synthesized in the rough endoplasmic reticulum, beginning with cholesterol as the precursor [235]. The final step in estrogen biosynthesis involves the aromatization of androstenedione to estrone or estradiol, catalyzed by the enzyme aromatase (CYP19A1). This process predominantly occurs in the ovarian granulosa cells, adrenal cortex, and adipose tissue, with smaller contributions from the breast and placenta. E4 synthesis, however, is restricted to the fetal liver during gestation [234]. Once synthesized, E2 circulates in the bloodstream bound to carrier proteins and diffuses across cell membranes to reach target tissues [236]. Its primary mode of action involves binding to nuclear estrogen receptors, forming complexes that regulate gene transcription either directly or through secondary signaling cascades. The biological effects of estrogens are influenced by the genetic and physiological context of the target cells. Although both sexes produce estrogens, their roles and concentrations differ. Beyond reproductive functions, estrogens are involved in metabolic regulation, energy homeostasis, and cardiovascular and neurological health. While they may offer cardioprotective benefits, they are also implicated in increased cardiovascular risk under certain conditions. Dysregulation of estrogen signaling is associated with various pathologies, including autoimmune, metabolic, and degenerative diseases, as well as hormone-sensitive cancers such as BCa [236].

**Figure 13:**
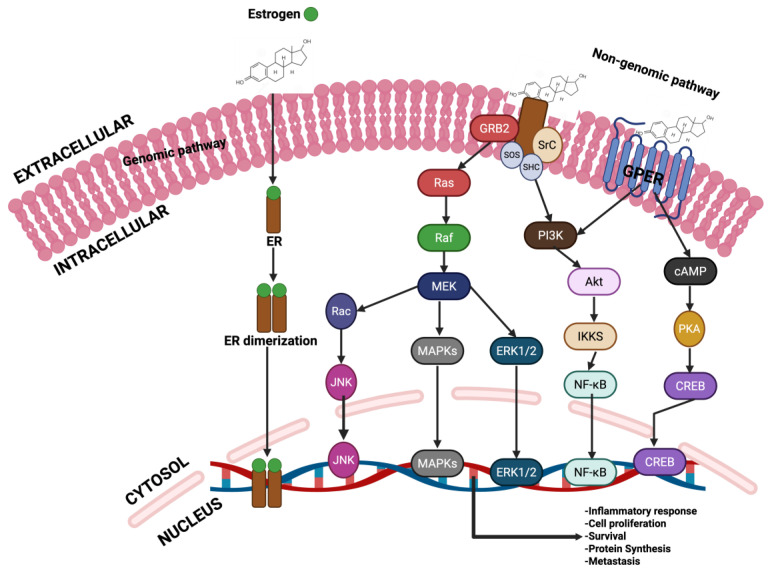
ER signaling pathway in BCa. The figure illustrates both genomic and non-genomic mechanisms of ER activation. In the genomic pathway, estrogen binds to nuclear ERs, leading to receptor dimerization and translocation into the nucleus, where it regulates gene transcription. In the non-genomic pathway, estrogen interacts with membrane-associated ERs, triggering rapid signaling cascades including Ras/Raf/MEK/ERK, PI3K/Akt, and Src-mediated pathways. These cascades promote cell proliferation, survival, inflammation, protein synthesis, and metastasis through downstream effectors such as JNK, p38 MAPKs, NF-κB, and CREB. Generated with BioRender (https://app.biorender.com, accessed on 22 June 2025).

ER signaling operates through both genomic and non-genomic mechanisms (**Figure 13**). In the genomic pathway, estrogen–ER complexes translocate to the nucleus and modulate gene expression. This can occur indirectly, where the ER interacts with DNA-binding transcription factors (TFs) at their response elements via protein–protein interactions, recruiting coactivators to influence transcription. For instance, ER can engage in non-classical signaling by interacting with transcription factors such as specificity protein 1 (Sp1) and nuclear factor kappa B (NF-κB), even in the absence of estrogen-response elements (EREs) in the target gene promoters [237,238]. In direct genomic signaling, ER binds directly to EREs in the promoter regions of target genes. Ligand binding induces a conformational change in the ER, facilitating its release from the heat shock protein 90 (HSP90) complex, a chaperone that stabilizes unbound ER and prevents its degradation [239]. There is substantial cross-talk between ER and other members of the steroid hormone receptor family, including progesterone receptors (PR), glucocorticoid receptors (GR), and AR, within BCa cells and other hormone-sensitive malignancies such as endometrial cancer [240]. These receptors exhibit overlapping DNA-binding motifs and coordinate their genomic functions through shared recruitment of transcriptional regulators and chromatin-modifying complexes [241]. ER signaling is modulated by the presence and activity of these other receptors. For instance, PR is not only a downstream target of ERα but also interacts directly with ERα, influencing its chromatin-binding landscape and thereby altering gene expression patterns that can impact clinical outcomes. Despite these observations, the exact molecular mechanisms by which PR reshapes ERα activity remain to be fully elucidated. Similarly, AR has been implicated in modulating ER genomic interactions in BCa and its functional role, along with therapeutic targeting strategies, has been investigated across various BCa subtypes [242].

In the context of non-genomic ER signalling, estrogen engages with membrane-associated estrogen receptors (mbERs) or G-protein–coupled estrogen receptor 1 (GPER1), initiating rapid signaling events at the cell surface independent of direct gene transcription [243]. These interactions activate intracellular kinase cascades, notably the PI3K/Akt/IKK/NF-κB and cAMP/PKA/CREB pathways. In addition, the binding of estrogen to ER can activate PI3K/Akt/IKK/NF-κB, Ras/Raf/MEK/MAPKs; MEK/Rac/JNK and MEK/ERK1/2 pathways. Within the MAPK cascade, estrogen binding activates Ras, which subsequently triggers Raf-mediated phosphorylation of MEK, leading to MAPK activation. MAPK then stimulates transcription factors such as c-Jun and c-Fos, promoting expression of downstream genes [244]. Also, mbER-mediated activation of SHC/PI3K/Akt initiates the PI3K–Akt–mTOR pathway, which regulates mTOR signaling. This pathway supports cellular proliferation and survival, and its dysregulation has been linked to the progression of ER-positive BCa and resistance to endocrine therapies [245].

The transcriptional activity of ER is tightly controlled by a network of coregulatory proteins, which include both coactivators and corepressors. These molecules are recruited to the ER via a conserved LxxLL motif within the ligand-binding domain (LBD), where “L” denotes leucine and “x” represents any amino acid [246]. Coregulators often function in concert with enzymatic partners such as methyltransferases, acetyltransferases, deacetylases, kinases, ubiquitin ligases, and ATP-dependent chromatin remodelers, thereby influencing chromatin structure and gene expression [247]. The dynamic balance between coactivators and corepressors is context-dependent and varies across cell types, ultimately shaping the transcriptional output of ER signaling. Corepressors, for example, recruit histone deacetylases (HDACs) to ER-bound chromatin, promoting chromatin condensation and transcriptional repression. By antagonizing coactivator function, corepressors help maintain appropriate levels of nuclear receptor activity. Aberrations in coregulator expression or function are strongly associated with tumorigenesis, cancer progression, and metastasis in malignancies such as breast and colorectal cancer [247].

#### Evidence of PFOS in Breast Cancer Development

**Figure 14:**
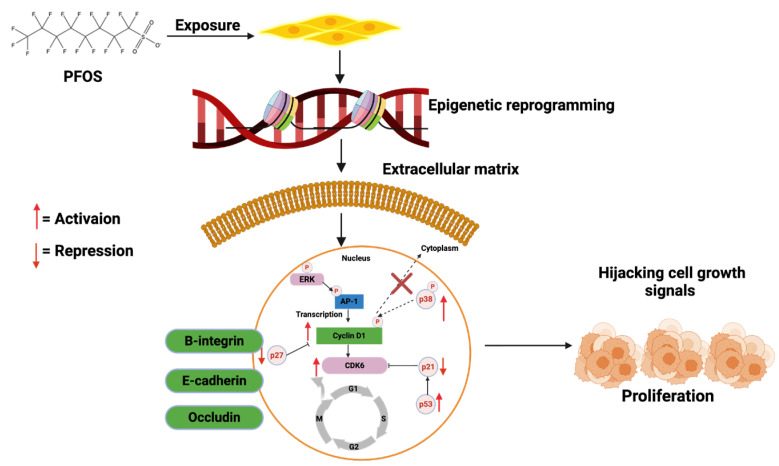
Mechanism of PFOS-mediated epigenetic perturbation in BCa. The figure illustrates how PFOS exposure induces epigenetic reprogramming through alterations in DNA methylation (5mC) and histone modifications. These changes disrupt the expression of genes regulating cell adhesion molecules such as β-integrin, E-cadherin, and occludin, compromising extracellular matrix integrity. The resulting epigenetic dysregulation hijacks cell growth signaling pathways such as ERK, AP-1, Cylin D1, and CDK4, promoting uncontrolled proliferation and contributing to BCa progression. Generated with BioRender (https://app.biorender.com, accessed on 29 October 2025).

Multiple studies have suggested a potential link between PFOS exposure and BCa development [248,249]. A case–control study conducted on the Greenland Inuit population found an association between elevated serum PFOS levels and an increased risk of BCa [26]. Additionally, PFOS is suspected to exhibit estrogenic properties, with in vitro studies indicating its ability to interact with ERα and ERβ, potentially enhancing ER-dependent transcriptional activity [31,250,251]. However, some studies have reported conflicting findings regarding its estrogenic effects, and the role of PFOS in BCa development remains insufficiently explored [252,253]. The findings by Pierozan et al. [254] found that PFOS led to a sustained rise in cyclin D1 levels, accounting for the increase in cell proliferation. Exposing MCF-10A cells to PFOS triggered the activation of ERK in unexposed daughter cells (**Figure 14**). ERK pathway activation is triggered by mitogen factors, and it is one of the most commonly mutated genes in various cancers, often resulting in increased cell proliferation [255]. Once activated, ERK translocates from the cytoplasm into the nucleus, where it phosphorylates various nuclear substrates, including transcription factors such as AP-1. AP-1 subsequently binds to the cyclin D1 promoter, thereby enhancing cyclin D1 gene transcription [256]. In another study, PFOS reduced the levels of the CDK inhibitor p21, whose loss is linked to enhanced tumorigenesis and poor prognosis across cancers [257,258]. PFOS also lowered p27 expression in D2 cells, a tumor suppressor frequently downregulated in BCa, where its loss predicts adverse clinical outcomes. Notably, p27 inactivation typically occurs through post-translational mechanisms such as degradation or mislocalization rather than mutation [259,260].

Metastasis remains the primary cause of mortality in BCa, occurring long after the initial tumor develops, with invasion into surrounding tissues as the first step [261,262]. The study of Pierozan et al. [254] indicated that PFOS promotes malignant transformation of MCF-10A cells by reducing the expression levels of key adhesion molecules, including occludin, E-cadherin, and β-integrins, thereby disrupting cell–cell and cell–matrix interactions. Such alterations are consistent with evidence that downregulation of E-cadherin facilitates BCa progression and metastasis, while reduced integrin expression impairs adhesion and drives tumor invasiveness [263,264]. Similarly, reduced occludin expression has been linked to metastatic BCa, while its overexpression inhibits tumour development and increases sensitivity to apoptosis [265,266].

Epigenetic signatures, such as DNA methylation, histone modification, and nucleosome remodelling, are now seen as crucial drivers of carcinogenesis [267,268]. Dysregulated epigenetic control in breast cells can alter gene expression, thereby contributing to the initiation and progression of BCa [269]. Exposure of PFOS to BCa cells increased the global DNA methylation in both MCF-10A cells and their unexposed progeny [254]. Such aberrant methylation patterns, particularly within CpG islands, can silence critical genes including tumor suppressors, DNA repair genes, and regulators of invasion, metastasis, and angiogenesis, thereby sustaining malignant transformation [270,271]. In cancer, CpG island hypermethylation is often coupled with histone H3 and H4 deacetylation, which alters chromatin architecture and disrupts gene expression [268,272]. Active transcription is generally associated with histone modifications such as H3K9ac, H3K27ac, and H3K4me, whereas H3K9 methylation promotes repressive chromatin states [270,273]. In their findings, PFOS decreased H3K9 acetylation, consistent with reduced acetylation observed in aggressive breast tumors and its correlation with poor prognosis [254]. These PFOS-induced histone modifications may contribute to dysregulation of cell-cycle regulators, as both promoter methylation and histone deacetylation are established mechanisms for p21 silencing [274].

Tsai et al. [275] identified a notable correlation between exposure to PFOS and BCa risk in Taiwanese women. Their case study revealed that women aged 50 or younger were particularly susceptible, with a stronger association observed in estrogen receptor (ER)-positive tumors within this age group. These results imply that younger women may be more sensitive to the carcinogenic effects of PFOS compared to older women. Supporting this, a study conducted in Greenland among women of similar ages (50 for cases and 54 for controls) also found a link between PFOS exposure and elevated BCa risk [26]. In contrast, a Danish study involving younger women (average age ~40 years) reported only a weak association between PFAS exposure and BCa [276]. BCa incidence is known to vary across age groups, ethnicities, and regions, likely due to differences in environmental exposures, reproductive patterns, and lifestyle choices [277]. While BCa risk typically increases with age in Western populations [278], this trend was not reflected in the Taiwanese cohort, where younger women showed higher vulnerability.

Previous studies have also suggested that PFOS may alter sex hormone levels, such as follicle-stimulating hormone (FSH) and testosterone, especially during adolescence (ages 12–17), a period marked by hormonal fluctuations [279]. Chronic exposure to PFOS could disrupt hormonal equilibrium, potentially contributing to BCa development [280]. Since their widespread use began in the 1950s, PFOS have been prevalent in industrial processes. Taiwanese environmental studies have detected elevated PFOS levels in rivers near industrial zones, indicating a possible route of exposure for nearby communities. This raises concerns about long-term exposure, particularly among younger individuals, and its implications for BCa risk. Their findings reinforced that women aged ≤50 years were more likely to develop ER-positive tumors in relation to PFOS exposure than those over 50. Similarly, Greenlandic research found a connection between PFOS and BCa risk in women of comparable age, although no significant difference was observed between ER-positive and ER-negative cases [281]. Conversely, a French study reported that low PFOS levels were associated with an increased risk of ER-negative BCa, with the average age at diagnosis being 68.8 years [50]. It is well-established that younger women diagnosed with BCa are more likely to present with ER-negative tumors [282]. While ER-positive BCa is often linked to reproductive factors, ER-negative variants are believed to be influenced more by environmental and lifestyle factors. Additionally, the classification of tumors as ER-positive or ER-negative may be affected by the timing of blood sample collection—whether before or after diagnosis [283]. Interestingly, PFOS concentrations in Taiwanese women were lower than those reported in studies from Greenland and France [50,281]. This discrepancy may be partially explained by age-related differences in tumor biology. Unlike Western trends, younger Taiwanese women (≤50 years) exhibited higher expression of estrogen and progesterone receptors compared to older women (>50 years) [277], a pattern also observed in other East Asian populations. While Wielsøe et al. [281] reported a positive association between PFOS and BCa risk, Bonefeld-Jørgensen et al. [276] did not find a significant link. Notably, Wielsøe et al. [281] documented higher PFOS concentrations than those observed in the current study. In contrast, Barry et al. [284] found that women with the highest PFOS levels had the lowest BCa risk, although their reported concentrations were also higher than those in the present research.

In animal models, mammary fibroadenomas have been observed following two years of PFOS exposure, leading the U.S. EPA to consider PFOS a possible BCa risk factor [285]. However, findings remain inconclusive. Hardisty et al. [286] observed no significant elevation in mammary gland tumors after a two-year dietary exposure to PFOS. Meanwhile, other research reported that perinatal exposure to PFOS in female mice caused persistent abnormalities and delayed mammary gland development [287], a phenomenon that may be mediated by activation of the PPAR-α pathway [288]. In addition, an in vitro study demonstrated that PFOS can enhance the activity of human BCa cells [289]. Although two studies have attempted to identify genes potentially affected by PFOS exposure in humans, the underlying mechanisms remain unclear [290]. Future research is therefore needed to investigate how PFOS may influence BCa pathogenesis in humans. Multiple recognized risk factors for BCa are associated with reproductive history, encompassing early menarche, late menopause, advanced age at first childbirth, breastfeeding practices, and the utilization of exogenous hormones [291]. Other lifestyle factors, including alcohol consumption, obesity, and lack of physical activity, also play a significant role [292]. Additionally, hereditary factors play a role in BCa incidence [293].

These studies are subject to several limitations. First, their cross-sectional nature prevents the establishment of a causal link between PFOS exposure and BCa risk. Second, the relatively small sample sizes contribute to statistical uncertainty, resulting in broad confidence intervals. Additionally, the analysis was restricted by age categorization and lacked sufficient data to thoroughly assess dose–response relationships. A major limitation was the absence of historical exposure records, which is critical given the long latency period associated with BCa development. Although PFOS compounds are known to persist in the body due to their long biological half-lives, relying on a single-time-point measurement may not accurately represent cumulative exposure. Despite these constraints, the studies (Table 1) offer valuable insights, particularly in highlighting age-specific associations between PFOS and BCa risk, which may be influenced by hormonal differences observed between Taiwanese and Western populations.

### 4.4. Ovarian Cancer: Mechanism and Carcinogenesis

**Figure 15:**
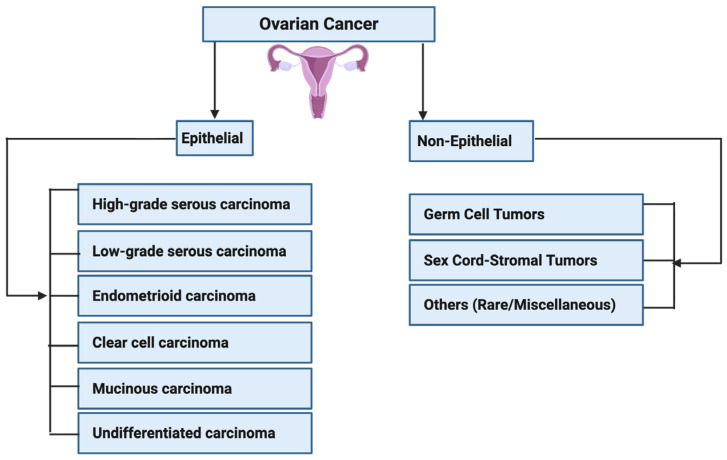
Histological subtypes of OC. Generated with BioRender (https://app.biorender.com, accessed on 29 October 2025).

OC is the second most common gynecologic disease in the United States. In 2024. There were about 19,680 diagnosed cases of OV and 12,740 related deaths worldwide [294]. OV is classified into two main histological subtypes (**Figure 15**): Epithelial ovarian cancers (EOCs) and non-epithelial ovarian cancers (non-EOCs). Approximately 90% of ovarian malignancies are EOCs, while the other 10% are non-EOCs. **Figure 15** shows the subtypes of EOCs and non-EOC [295]. OC is broadly classified into epithelial and non-epithelial types. Epithelial ovarian cancers (EOCs), which represent the majority of cases, include high-grade serous carcinoma, low-grade serous carcinoma, endometrioid carcinoma, clear cell carcinoma, mucinous carcinoma, and undifferentiated carcinoma. Non-epithelial ovarian cancers (non-EOCs) include germ cell tumors, sex cord–stromal tumors, and other rare or miscellaneous tumor types. The molecular biology of OC is complex and involves several hormonal, genetic and epigenetic modifications that promote its tumorigenesis and the development of treatment resistance.

Several studies indicated that hormonal factors play a crucial role in the tumorigenesis and progression of different OC subtypes [296,297,298]. For example, it has been shown that hormonal factors, particularly estrogens, play a crucial role in the development and progression of EOCs. Estrogens primarily affect the proliferation, invasion and epithelial-to-mesenchymal transition of cancer cells through their effects on ER including ERα, ERβ, and GPER1. Furthermore, estrogen signaling affects the tumor microenvironment by modulating immune and stromal cells, which further promotes tumor progression and invasion [299]. EOCs, especially the high-grade serous ovarian carcinoma (HGSC) type, tend to be more aggressive and have worse survival rates if diagnosed at advanced stages. It is now known that the fallopian tube epithelium, particularly the fimbrial end rather than the ovarian surface is the primary source of HGSC. This shift is supported by molecular and histopathological evidence identifying precursor lesions such as p53 signatures and serous tubal intraepithelial carcinoma (STIC), which represent early stages in the progression to invasive cancer. Especially among women with BRCA mutations, occult tubal lesions are frequently found during prophylactic surgeries, reinforcing the tubal origin hypothesis [300].

Although several genes and transcription factors are involved in the development and progression of HGSC, the *BRCA1* and *BRCA2* genes are considered the most significant contributors. Homologous recombination (HR) is one of the DNA repair mechanisms that repairs the double-strand breaks by using an intact homologous DNA molecule as a template. Mutations or epigenetic silencing of BRCA1/2 compromise the HR repair mechanism, leading to genomic instability and tumorigenesis. About 50% of high-grade serous ovarian cancers (HGSOC) exhibit homologous recombination repair (HHR), with approximately 15–20% harboring germline or somatic mutations in *BRCA1* or *BRCA2*. The identification of HRR deficiencies has thus become a crucial factor in guiding precision medicine strategies targeting OC. These HRR-deficient tumors are particularly sensitive to DNA-damaging agents like platinum-based chemotherapies and to targeted therapies such as poly (ADP-ribose) polymerase (PARP) inhibitors. PARP inhibitors (PARPi) such as Olaparib, Niraparib and Rucaparib exploit the concept of synthetic lethality by blocking alternative DNA repair pathways, leading to the accumulation of DNA damage and cell death in HRR-deficient cancer cells. Since its first approval in 2014, PARPi has revolutionized OC care with dramatic improvements in progression-free and overall survival when used as maintenance therapy following platinum-based chemotherapies [301,302]. In recent years, a significant challenge in OC has emerged that has raised our concerns. OC cells show resistance mechanisms to PARPi and chemotherapeutics. This phenomenon has led to a decline in overall survival and progression-free survival rates among patients [301]. Resistance to PARP inhibitors in OC can occur through restoration of homologous recombination (e.g., BRCA reversion mutations), replication fork protection, PARP1 mutations, and increased drug efflux [301,302]. On the other hand, Cisplatin resistance involves enhanced DNA repair, reduced drug accumulation, detoxification by glutathione, and evasion of apoptosis [303].

#### Evidence of PFOS in Ovarian Cancer Development

Chronic low-dose exposure to PFOS in female mice has been shown to alter steroidogenesis and hormonal regulation, consequently disrupting reproductive function. PFOS lengthens the estrous cycle, especially extending the diestrus phase, and reduces the number of mature ovarian follicles and corpora lutea while increasing follicular atresia. These alterations are accomplished by a drop in the serum levels of important respective hormones, including LH, FSH, P4, E2 and GnRH. Mechanistically, PFOS suppresses the expression of the steroidogenic acute regulatory protein (StAR) gene in the ovary by reducing histone H3K14 acetylation at its promoter, leading to diminished estrogen biosynthesis. The underlying cellular mechanism involves a reduction in histone H3K14 acetylation at the steroidogenic acute regulatory protein, which leads to inhibition of estrogen biosynthesis, impaired follicle development and ovulation, thereby contributing to a reduction in ovarian follicular reserve [304]. PFOS is known to bind to ER, thereby partially blocking and activating their responses [305]. This interference creates a restriction in regulating the genome and therefore increases the risk of OC. The mechanistic pathway involves GPR30 and the Insulin-like growth factor 1 receptor (IGF1R), a growth-promoting factor found in granulosa cells that responds to PFOS in human follicular fluid, thereby promoting the growth of ovarian granulosa tumours [306].

This epigenetic modification results in impaired follicle maturation, disrupted activation of kisspeptin neurons in the anteroventral periventricular nucleus (AVPV), and a blunted LH surge necessary for ovulation. Notably, administration of estrogen or kisspeptin-10 can rescue the impaired LH surge, highlighting the central role of hormonal signaling pathways affected by PFOS. Also, recent findings showed that exposure to PFOS negatively impacts uterine health by dropping the viability of human endometrial stromal cells, causing apoptosis through the upregulation of BAX and the downregulation of BCL-2, interfering with the progression of the cell cycle, and changing receptivity markers like HO, XA10, ITGB3, and FOXO1 [307]. These results imply that PFOS alters the hormonal regulation of reproduction and mitochondrial integrity factors that also play a pivotal role in OC development. The cellular stress, endocrine disruption, and inflammatory environment triggered by PFOS in uterine tissues may extend to ovarian tissue, potentially increasing the consequences of transformation into cancer. Like PFOS, other PFAS, including PFOA, PFHpA, and PFPA, have been found to increase the resistance of OC cells to various treatments, including chemotherapy. Notably, these PFAS at environmentally relevant, sub-cytotoxic concentrations enhanced OC cell survival and increased resistance to the chemotherapy drug carboplatin [308].

Increasing evidence (Table 1) has reported that exposure to PFOS could adversely affect key processes like ovarian folliculogenesis and steroidogenesis, which are needed for normal reproductive health. Epidemiological evidence has reported that exposure to PFOS impacts ovarian steroidogenesis, causes a delayed onset of menarche, disrupts the menstrual cycle, accelerates ovarian ageing, and may cause chronic conditions such as polycystic ovarian syndrome (PCOS) and increases the chances of OC [309]. The study conducted by Knox et al. [310] has reported PFOS exposure to have a significant and negative relationship with serum E2 levels among women aged 42-65 years. Similarly, Zhang et al. [311] suggested that exposure to PFOS may lead to decreased serum E2 and prolactin levels, thereby causing an increase in FSH levels among premature ovarian insufficiency in patients. Their study also revealed higher plasma PFOS concentrations, indicating an increased risk of POI. PFOS has been reported to have weak estrogenic activity, whereas it exerts an anti-estrogenic effect when co-administered with estradiol. The result showed a negative association between PFOS and E2 and a positive association with FSH. PFOS has been reported to cause a decrease in the production of estradiol in women of reproductive age and to alter steroidogenesis. Higher exposure to PFOS has been reported to cause a reduction in the ovarian follicular reserve. The study conducted by Jones et al. [312] found a positive association with OC, revealing an increased risk of serum levels of certain PFOS, which are associated with an increased risk of OC among postmenopausal women. Their study measured the amount of PFOS in blood samples collected before developing OC. After analysing data from 318 OC cases and 472 matched controls, the result showed 47% odds of developing OC.

**Table 1:** Summary of Research Findings on PFAS and Cancer.

S/N	Study/Model	Findings	References
1.	Human prostate stem/progenitor cell (SPC) population	PFOS increased carcinogenic risk, tumor progression and spheroid formations in the prostate gland as well as increased the expression of PPARα and RXRα.	Wen et al. [211]
2.	Epidermal pre-malignant stem cells	PFOS upregulated serine and glycine metabolism and increased the growth of PCa.	Imir et al. [226].
3.	Case–control study	Significant association between elevated serum PFOS levels and an increased risk of BCa	Bonefeld-Jorgensen et al. [26].
4.	Juvenile rainbow trout (Oncorhynchus mykiss)	PFOS could interact with ERα and ERβ, potentially enhancing ER-dependent transcriptional activity	Benninghoff et al. [250]
5.	H295R cells	PFOS serves as an ER agonist and Thyroid hormone receptor (THR) antagonist, as well as increased estradiol (E2) levels in H295R cells.	Du et al. [31]
6.	MVLN cells	PFOS significantly induced the ER transactivity while antagonizing the activity of AR.	Kjeldsen et al. [251]
7.	MCF-10A	PFOS elevated cyclin D1 and D2 levels, increased the global DNA methylation, as well as reduced the levels of the CDK inhibitor p21, occluding, E-cadherin and β-integrins.	Pierozan et al. [254]
8.	Case–control study	Women aged 50 or younger were particularly susceptible to PFOS, with a stronger association observed in estrogen receptor (ER)-positive tumors within this age group.	Tsai et al. [275]
9.	Case–control study	There is a strong link between PFOS exposure and elevated BCa risk.	Bonefeld-Jorgensen et al. [26].
10.	Case–control study	PFOS triggered hormonal fluctuations in adolescents aged 12–17 years.	Tsai et al. [275].
11.	Case–control study	Significant increase in PFOS levels in cases compared to the controls, indicating the correlation of PFOS with BCa.	Wielsøe et al. [281]
12.	T47D human BCa cells	PFOS promoted the estrogenic effects of 17β-estradiol in T47D human BCa cells.	Sonthithai et al. [289]
13.	human endometrial stromal cells (hESCs)	PFOS may drive OC by decreasing the expression levels of endometrial tolerance-related proteins Homeobox A10 (HOXA10) and integrin beta 3 (ITGB3), while increasing the expression level of Forkhead box 01 (FOXO1) protein.	Ren et al. [307].
14.	Age-Related Associations	PFOS revealed a positive association with PCa in men aged ≥ 70 years.	Alyssa et al. [216]
15.	Chinese women study	PFOS exposure decreased serum E2 and prolactin levels and increased FSH levels, disrupted ovarian steriodogenesis, and caused premature ovarian insufficiency	Zhang et al. [311]
16.	OC risk	PFOS exposure is associated with OC incidence.	Jones et al. [312]
17.	Sex Hormones	PFOS significantly impacted the serum E2 levels among women aged 42–65	Knox et al. [310]

## 5. Regulatory Actions Against PFOS

Due to the recognized environmental and health hazards posed by PFOS, regulatory bodies, particularly in industrialized nations, have enacted stringent controls on its manufacture and use. In 2009, PFOS was added to the Stockholm Convention on Persistent Organic Pollutants, prompting a global phase-out coordinated by the United Nations Environment Program (UNEP) [313]. Additionally, the U.S. Food and Drug Administration (FDA) withdrew approval for PFOS and several other PFAS in food contact materials. Ongoing investigations by the Agency for Toxic Substances and Disease Registry (ATSDR), under the Centers for Disease Control and Prevention (CDC), are examining human exposure routes and health outcomes linked to PFOS and related PFAS, particularly through drinking water and dietary intake. According to the U.S. Environmental Protection Agency (EPA) and the National Primary Drinking Water Regulation (NPDWR), the enforceable Maximum Contaminant Level (MCL) for PFOS in drinking water is set at 4.0 parts per trillion (ppt), equivalent to 4.0 ng/L [314]. The European Union (EU) has similarly imposed strict limitations under the REACH (Registration, Evaluation, Authorization and Restriction of Chemicals) framework, promoting the development and adoption of safer chemical alternatives [315].

Recently, the International Agency for Research on Cancer (IARC) classified PFOS as a Group 2B substance, indicating it is possibly carcinogenic to humans. This classification is based on evidence linking prolonged PFOS exposure to various cancers, including those of the pancreas, breast, prostate, liver, kidney, ovary, endometrium, bladder, thyroid, and non-Hodgkin lymphoma [32,51,91,128,316,317,318,319]. Although animal studies provide limited carcinogenic data, research by Tessmann et al. [320] demonstrated that PFOS exposure in mice led to the suppression of 3-hydroxy-3-methylglutaryl-CoA synthase-2 and the activation of oncogenic proteins such as c-MYC, FASN, mTOR, and β-catenin—markers associated with intestinal tumorigenesis.

While developed countries have made substantial progress in regulating PFOS and other hazardous chemicals, many developing and underdeveloped nations still lack comprehensive frameworks to mitigate environmental contamination. As highlighted by Wee and Aris, these regions often face systemic challenges, including the absence of standards for physical water parameters (e.g., pH, turbidity), insufficient regulation of chemical contaminants (e.g., disinfectants, heavy metals, nutrients), inadequate monitoring of organic pollutants (e.g., pesticides, petroleum derivatives), and limited capacity to assess microbiological indicators (e.g., coliforms, Escherichia coli) and sensory attributes (e.g., taste, color, odor, texture, temperature, and overall acceptability) [321]. Despite some progress, such as the establishment of water quality standards in Malaysia, India, Bangladesh, the Philippines, China, Turkey, the United Arab Emirates, South Africa, Nigeria, Ghana, and others, PFOS continues to pose exposure risks to both humans and wildlife due to its persistence and widespread distribution in the environment.

## 6. Conclusions

PFOS, a persistent organic pollutant, presents a substantial risk to environmental and human health due to its chemical stability, bioaccumulative properties, and resistance to degradation. This review has outlined PFOS’s complex toxicological profile, emphasizing its widespread environmental dissemination via both point and nonpoint sources, its intricate pharmacokinetics, and its limited excretion pathways. Mechanistic studies reveal that PFOS disrupts lipid metabolism, induces oxidative stress, impairs mitochondrial function, and interferes with endocrine and immune signaling. These molecular perturbations are implicated in a range of adverse health outcomes, including hepatotoxicity, neurotoxicity, reproductive dysfunction, and immunosuppression. Notably, emerging evidence suggests a potential role for PFOS in carcinogenesis, particularly in hormone-sensitive cancers such as prostate, breast, and ovarian cancers. Proposed mechanisms include endocrine disruption, oxidative DNA damage, chronic inflammation, and epigenetic alterations. Epidemiological and experimental data increasingly support associations between PFOS exposure and elevated cancer risk, especially among populations with occupational or environmental exposure. PFOS has been shown to activate oncogenic pathways, modulate stem cell behavior, and promote tumor growth and metastasis. These findings underscore the need for continued investigation into PFOS’s molecular mechanisms and long-term health effects. Although regulatory measures in several developed countries have curtailed PFOS production and usage, many developing regions lack comprehensive frameworks to monitor and mitigate its impact. Addressing the global burden of PFOS requires coordinated international efforts to enhance surveillance, enforce stringent regulations, and accelerate the development of safer chemical alternatives.

## Abbreviations

AVPV	Anteroventral Periventricular Nucleus
AFFF	Aqueous Film Forming Foam
BAX	BCL2 Associated X Protein
BCL-2	B-Cell Lymphoma 2
BRCA1	Breast Cancer 1 Gene
BRCA2	Breast Cancer 2 Gene
cAMP	Cyclic Adenosine Monophosphate
CDK	Cyclin-Dependent Kinase
CREB	cAMP Response Element-Binding Protein
CYP	Cytochrome P450 (CYP450)
DNA	Deoxyribonucleic Acid
E2	Estradiol
ER	Estrogen Receptor
ERK	Extracellular Signal-Regulated Kinase
FOXO1	Forkhead Box Protein O1
FSH	Follicle-Stimulating Hormone
FSH.	Same as above (Follicle-Stimulating Hormone)
GnRH	Gonadotropin-Releasing Hormone
GPR30	G Protein-Coupled Estrogen Receptor 30 (also known as GPER1)
GSH	Glutathione
GSK-3β	Glycogen Synthase Kinase 3 Beta
GST	Glutathione S-Transferase
H3K14	Histone H3 Lysine 14 (site of acetylation or methylation)
HDACs	Histone Deacetylases
HER2	Human Epidermal Growth Factor Receptor 2
HO	Heme Oxygenase
HO-1	Heme Oxygenase-1
IARC	International Agency for Research on Cancer
IGF1R	Insulin-Like Growth Factor 1 Receptor
ITGB3	Integrin Beta 3
JNK	c-Jun N-terminal Kinase
LBD	Ligand-Binding Domain
LH	Luteinizing Hormone
MAPKs	Mitogen-Activated Protein Kinases
MCF-10A	Human Non-Tumorigenic Mammary Epithelial Cell Line
MMP	Matrix Metalloproteinase
NF-κB	Nuclear Factor Kappa-Light-Chain-Enhancer of Activated B Cells
NF-κB,	Same as above
p38	p38 Mitogen-Activated Protein Kinase (MAPK)
P4	Progesterone (also known as Pregn-4-ene-3,20-dione)
PARP	Poly (ADP-Ribose) Polymerase
PFAS	Per- and Polyfluoroalkyl Substances
PFHpA	Perfluoroheptanoic Acid
PFOA	Perfluorooctanoic Acid
PFOS	Perfluorooctane Sulfonate
PFPA	Perfluoropentanoic Acid
PPARα	Peroxisome Proliferator-Activated Receptor Alpha
PIN	Prostatic Intraepithelial Neoplasia
ATP	Adenosine Triphosphate
PKA	Protein Kinase A
POI	Primary Ovarian Insufficiency
PR	Progesterone Receptor
PSA	Prostate-Specific Antigen
PTEN	Phosphatase and Tensin Homolog
ROS	Reactive Oxygen Species
StAR	Steroidogenic Acute Regulatory Protein
TNBC	Triple-Negative Breast Cancer
VEGF	Vascular Endothelial Growth Factor
WNT1	Wingless-Type MMTV Integration Site Family Member 1
XA10	Xanthine Dehydrogenase/Aldehyde Oxidase 1 (also known as xanthine oxidase homolog, depending on context)
